# The relative weight of ontogeny, topology and climate in the architectural development of three North American conifers

**DOI:** 10.1093/aobpla/ply045

**Published:** 2018-07-31

**Authors:** Fabien Buissart, Michel Vennetier, Sylvain Delagrange, François Girard, Yves Caraglio, Sylvie-Annabel Sabatier, Alison D Munson, Eric-André Nicolini

**Affiliations:** 1 Irstea UR RECOVER/Ecosystèmes Méditerranéens et Risques, Centre d’Aix-en-Provence, Aix-En-Provence Cedex, France; 2 Aix-Marseille Université, Jardin du Pharo-58, bd Charles Livon, Marseille Cedex, France; 3 ECCOREV FR 3098, Technopôle de l’environnement Arbois-Méditerranée, Domaine du Petit Arbois, Avenue Louis Philibert, Bâtiment du CEREGE BP, Aix-en-Provence cedex, France; 4 Institute of Temperate Forest Sciences (ISFORT), University of Quebec in Outaouais (UQO), Rue Principale, Ripon, Québec, Canada; 5 Université de Montréal (UM), 520 chemin de la Côte-Ste-Catherine, Montréal, Québec, Canada; 6 CIRAD, UMR AMAP, Montpellier, France; 7 Université Laval, Centre d’étude de la forêt, Faculté de foresterie, de géographie et de géomatique, rue de la Terrasse, Québec, Québec, Canada

**Keywords:** Canada, climate, ontogeny, PLS regression, Québec, *Picea mariana*, *Pinus banksiana*, *Pinus strobus*, topology, tree architecture

## Abstract

Knowledge of plant architecture allows retrospective study of plant development, hence provides powerful tools, through modelling and simulation, to link this development with environmental constraints, and then predict its response to global change. The present study aims to determine some of the main endogenous and exogenous variables driving the architectural development of three North American conifers. We measured architectural traits retrospectively on the trunk, branches and twigs of whole tree crowns for each species: annual shoot length (ASL), needle length, branching patterns and reproduction organs (male and female). We fitted a partial least square (PLS) regression to explain each architectural trait with respect to topological, ontogenic and climatic variables. Results showed a significant weight of these three groups of variables for previous and current year, corresponding, respectively, to organogenesis and elongation. Topological and ontogenic variables had the greatest weight in models. Particularly, all architectural traits were strongly correlated with ASL. We highlighted a negative architectural response of two species to higher than average temperatures, whereas the third one took advantage of these higher temperatures to some degree. Tree architectural development weekly but significantly improved with higher precipitation. Our study underlines the strong weight of topology and ontogeny in tree growth patterns at twig and branch scales. The correlation between ASL and other tree architectural traits should be integrated into architectural development models. Climate variables are secondary in importance at the twig scale. However, interannual climate variations influence all axis categories and branching orders and therefore significantly impact crown development as a whole. This latter impact may increase with climate change, especially as climate affects architectural traits over at least 2 years, through organogenesis and elongation.

## Teaser

This study proposes an original insight into tree architectural development, by combining in a simultaneous analysis three sets of explanatory variables: ontogenic, topological and climatic factors. It targets three common North American conifers (*Picea mariana*; *Pinus banksiana*; *Pinus strobus*), the detailed architecture of which is poorly known. Thus it brings new knowledge about these species and their response to environmental constraints. Our results may help improve the assessment of the future of these species in the context of climate change, particularly through the knowledge of interactions between exogenous (climate and site conditions) and endogenous factors (ontogeny, topology).

## Introduction

Climate, as more generally environmental conditions, impacts plant growth and consequently plant architecture. The manner tree architecture regulates phenotypic plasticity is a specific response. Kunstler *et al.* (2016), through a global approach, suggest that some architectural traits are useful to model tree-crown competition and resulting forest communities. Understanding the response of tree architecture to climate variability is also challenging, but necessary to better assess and simulate forest response to climate change.

Primary growth, driving architectural development in plants, corresponds to the creation of new tissues outside existing organs. For a tree, it includes growth length of existing axes (bole, branches and roots), branching processes (formation of new branches or roots), genesis and growth of leaves, needles and rootlets, and reproduction (flowering, fruiting) (Hallé *et al.* 1978; Barthélémy and Caraglio 2007).

Spatial organization of axes (topology) combined with its changes over time (ontogeny) defines plant architecture. Plant architecture is the expression of a balance between endogenous, genetically or physiologically driven growth processes, and exogenous environmental constraints. Plant architectural analysis provides a comprehensive approach to understand plant development, with applications in many plant biology disciplines (Kaplan 2001). Describing and analysing tree architecture is a challenge: on the one hand, because of tree size, subsampling and destructive methods are required (Barthelemy *et al.* 1989); on the other hand, the long life span of trees allows numerous endogenous and exogenous factors to interact with tree architecture. Indeed, tree development patterns change with age, life stages (Chaubert-Pereira *et al.* 2009), competition or accidents such as wind or snow breaks, herbivory, climate extremes (Poethig 1990; Barthélémy and Caraglio 2007), and potentially with climate change in the long term (Girard *et al.* 2011; Girard *et al.* 2012).

A coarse representation of tree crown architecture at different scales is widely used as a tool to study the forest canopy, for example, to assess light interception (Honer 1971; Fournier *et al.* 1996), to support remote sensing models (Landry *et al.* 1997) or to model forest vulnerability to fire (Caraglio *et al.* 2007; Cruz and Alexander 2012). Tree architecture also provides useful information to study carbon partitioning within trees (Taugourdeau *et al.* 2012), to build forest management scenarios (Courbaud *et al.* 2001) or to diagnose tree health status (Innes 1998; Dobbertin 2005; Sabatier *et al.* 2014).

But except for tree height, few papers investigated the relationships between primary growth and climate variability (Thabeet *et al.* 2009; Girard *et al.* 2012; Vennetier *et al.* 2013). Such information is required to improve tree growth and architecture modelling, and is critical to accurately simulate future tree growth in the context of global change. Most existing tree development models use mean parameters averaged over entire branches or trees (Barczi *et al.* 2008; Buck-Sorlin *et al.* 2008), even though architectural traits of branch axes, as well as their response to climate, differ according to their position in the crown and in the branching hierarchy (Girard *et al.* 2011).

Cold, including extreme frost and a short growing season, limits tree growth in high latitudes (Gamache and Payette 2004), and particularly in boreal forests (Plasse and Payette 2015). Increases in forest productivity may occur as climate warming allows a potentially longer growing season in cold-limited environments (Chuine and Beaubien 2001). But the positive effects of warming leading to advanced phenology in spring, and delay in autumn dormancy, can be offset by local adaptation of species, depending on factors such as photoperiod, chilling requirements, vernalization time or interactions with soil fertility and especially water status. Such offsets have been demonstrated at local to large scales under natural conditions (Johnsen *et al.* 1996; Creed *et al.* 2015), in controlled environments (Li *et al.* 2015) and by modelling (Clark *et al.* 2014).

As an illustration of such limitations, recent studies reported cases of warming-induced decreases in tree growth: negative correlations were found between ring-width and warmer than average temperatures for several North American species, including black spruce—*Picea mariana* (Walker and Johnstone 2014) and eastern white cedar—*Thuja occidentalis* (Housset *et al.* 2015). Some of the responses present a north-south contrast logically related to a temperature gradient, with summer temperatures more important at the higher latitudes for black spruce, and east-west contrasts linked to a humidity gradient (Nicault *et al.* 2014).

The aim of the present study was to understand the relationship between architectural development and a set of endogenous (ontogenic, topological) and exogenous (climate, site condition) variables for three frequent species of Canadian forests—black spruce, Jack pine (*Pinus banksiana*) and Eastern white pine (*Pinus strobus*). While plant architecture is the result of meristem genetic regulation (Chomicki *et al.* 2017) and can be useful to infer physiological mechanisms (i.e. Peyhardi *et al.* 2017), in this study we did not aim to further investigate the ecophysiological processes behind architectural development. We rather aimed to answer the following questions: (i) What are the interactions among measured architectural variables? (ii) How does interannual climate variability affect tree architectural development? (iii) What is the relative influence of climate variability, compared to ontogenic and topological factors in driving tree architectural development?

## Methods

### Study sites

**Figure 1.**
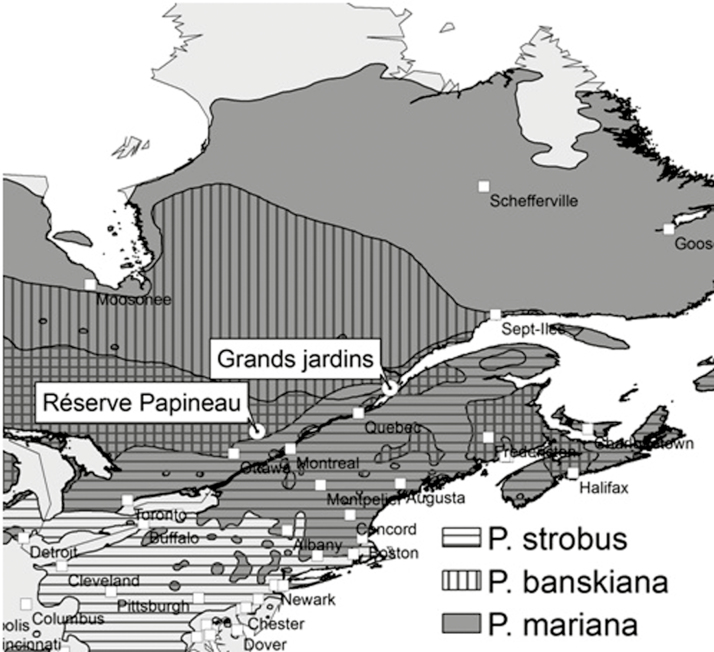
Map locating study sites and the range of studied species within the study area.

Study sites included two mature natural forests in southern Québec (Canada): ‘Réserve Papineau-Labelle’ (studied species—Eastern white pine) and ‘Parc des Grands Jardins’ (studied species—black spruce and Jack pine). The location of both sites and the range of each studied species are shown in Fig. 1.

The Réserve Papineau (46°08′48″N, 75°09′49″W) is a temperate forest dominated by *Acer saccharum* and *Betula alleghaniensis*, accompanied by several species of oak, beech, linden and ash, and scattered stands of Eastern white pine. In our three plots, these pines were 87-year-old (SD 17 years), with mean diameter and height of, respectively, 38 cm (SD 5.8 cm) and 21.5 m (SD 4.2 m). This location, with elevation ranging between 200 and 500 m, has a cool temperate climate (Rizzo and Wiken 1992), with a mean annual temperature of 5.2 °C and annual precipitation of 924 mm (2000–13 data from Chénéville weather station, 45°53′22,762″N, 75°03′36,421″W; Environnement Canada 2016). The mineral soil is a sandy loam, overlain by a shallow layer of organic matter. The three plots mainly differ based on their slope and composition of the soil coarse fraction. The first plot is located at the bottom of a hill (slope between 0 and 5 %) with absence of rocks. The second is at the middle of a gentle slope (5–15 %) with presence of some scattered rocky outcrops, and the third is on the upper part of a steep slope (20–30 %) with large and numerous rocky elements.

The Parc des Grands-Jardins (PGJ) is located in the Charlevoix highlands, about 120 km north-east of Quebec City (Fig. 1). The area is characterized by high hills with an average altitude between 700 and 900 m above sea level (a.s.l.), with several alpine and subalpine summits over 1000 m a.s.l. The average annual temperature is about 0 °C, the mean temperature of the warmest month (July) is 15 °C, and the average frost-free period of 60–70 days is among the shortest in southern Québec. Meteorological data from L’Étape Station located at 47°33′44″N, 71°13′44″W (1999–2013) was the closest available. A strong precipitation gradient exists between L’Étape (1963–2013) and the PGJ former station (La Galette 47°43′52″, 70°43′31″; 1963–74). The gradient represents a decrease of 33 % of annual precipitation in the PGJ that was accounted for in the calculations and interpretation of results. The vegetation in the PGJ forms a mosaic of open and closed forests, where black spruce (*P. mariana*) is the dominant tree species. Eastern larch (*Larix laricina*), balsam fir (*Abies balsamea*) and paper birch (*Betula papyrifera*) are growing in moderately to poorly drained sites preserved from extensive logging. The black spruce forests in the PGJ area are found on well-drained, acidic podzol soils of the Malbaie River Basin. The soils are composed of fluvio-glacial sands and gravel in valleys and heterometric till on slopes. Conifer forests in the Park are subject to several disturbances, including insect outbreaks (spruce budworm, *Choristoneura fumiferana*) and fires. Several forest tracts were also harvested before the creation of the Park in 1981. Three recorded insect outbreaks occurred during the 20th century, with the last one extending from the mid-1970s to the mid-1980s (Boisclair 1990).

### Data acquisition

We sampled 15 dominant trees per species, free from any significant competition with neighbours. Each tree consisted of one dominant main axis (trunk = order 1) bearing a crown made of lateral branches (order 2). For each tree of black spruce and Jack pine, we collected three coupled branches, respectively, in the top, middle and low crown, with opposite orientations within a couple (Girard *et al.* 2011). For Eastern white pine, due to the impossibility to harvest trees, no branches from the very top crown could be collected, and they were replaced by an additional pair of branches as high as possible in the middle of the crown. We also collected the whole top of the trunk and crown of three black spruce trees and one Jack pine. Tree age was estimated by counting rings from a core at breast height.

Architectural and growth measurements were performed in the lab. Tree cores were glued to a support and sanded. Then, ring-width was measured using WinDendro (Guay 2012); cross-validation and standardization were carried out according to standard procedures (Payette and Filion 2010). We considered the main axis of each trunk and branch, and for each main branch axis, two to three evenly distributed opposite couples of their twigs (branching order 3). Morphological markers like size and density of scales at the base of the growth units, lateral axes and position of cones were used to delimit growth units and annual shoots (one annual shoot can be composed of several growth units for polycyclic species). These markers allowed us to reconstruct axis primary growth for the last 12–15 years (markers fade and are no longer reliable for older shoots), following methods described by Sabatier *et al.* (2003) and Pardos *et al.* (2003). Then for selected axes, we considered the following dependant (Table 1) and explanatory variables (Tables 2 and 3) for each annual shoot.

**Table 1.** Dependent architectural variables. *The number of branches per annual shoot or growth unit is thereafter named branching rate.

Variable	All variables refer to a single annual shoot or growth unit
ASL	Annual shoot length
NL	Mean needle length
N ram*	Number of branches (ramifications) for pine species
whorl ram*	Number of branches of the pseudowhorl for black spruce
int ram*	Number of interwhorl branches for black spruce
Polyc	Polycyclism rate for pines
Lg ♂	Length of male cones (Barthélémy and Caraglio 2007), for pines
P ♂	Presence/absence of male cones (binary)
N ♂	Number of male cones, for the black spruce
N ♀	Number of female cones

**Table 2.** Independent explanatory variables: topological variables. **Vigour index (ranging from 0 to 1 from the weakest to the strongest) is the probability of the value of the axis growth pattern for the age of the annual shoot in the normal distribution of shoot length values for this age within its hierarchical order.

Variable	Description
Order	Three values: 1 = trunk, 2 = main axis of sampled branches, 3 = first level branching (twigs) of branch main axis
Vigour**	Relative vigour index of the axis: computed for each axis from its shoot length growth pattern **[see****Supporting Information—Appendix 1****]**.

**Table 3.** Independent explanatory variables: ontogenic variables.

Variable	Description
Age	Ontogenic age = rank of an annual shoot relative to the first annual shoot at the base of the axis (Barthélémy and Caraglio 2007)
Tree age	Age of the tree at the year of a given annual shoot development
Autocorrelation	Value of the previous year (*n* − 1) for each architectural variable

Annual shoot length (ASL) is by itself a dependent variable, but we used it as an explanatory factor for other architectural variables. This does not induce any circularity in the analyses. Although ASL is highly dependent on most topological and ontogenic factors, axis vigour is usually considered one of the main drivers of tree crown development (Thabeet *et al.* 2009; Girard *et al.* 2011,^2012^). Independently from ASL, this development also partially depends on the same topological and ontogenetic factors. The relative weight and role of axis vigour, axis hierarchy and branch position in the crown was disentangled by Vennetier *et al.* (2013), as well as in the above-mentioned references, validating the method used here. Moreover, the statistical method used for the analyses (partial least square (PLS) regression) is designed to free the results from the interactions between correlated variables.

Local variables (sites, trees and branches, climate)

The autocorrelation aimed to determine the relative weight of climate variability, heritable vigour related to tree and branch health status, and local invariable (site) or more slowly changing ontogenic and topological variables (related to individual trees and branches). The relative vigour was used with the same goal, but was focused to more precisely analyse the role of hierarchical branching order.

Local variables accounted for the portion of variability in architectural variables related to: (i) site effect—differences in soil fertility and topography among sites; (ii) tree effect—individual tree characteristics linked to genetics or growth history, and to local variations of soil and topographic conditions within a site; and (iii) branch effect—individual branches that differ according to their position in the crown (top, middle, low; Thabeet *et al.* 2009; Girard *et al.* 2011, 2012). We eliminated biotic effects of competition and insects on branches by selecting those with minimal competition and no apparent past accidents (breakage or other damage).

From raw data of the weather stations, we computed seasonal, monthly and bimonthly rainfall, temperature and degree-days (DD) for each site. Groups of 3 months were related to the main corresponding season: winter (January–March), spring (April–June), summer (July–September) and autumn (October–December).

### Statistical analyses and modelling

All the analyses were performed with R statistical software (version 2.15.1; R Core Team 2014). To each annual shoot, and its architectural and growth parameters, we associated climatic variables of both the year of elongation (year *n*) and the year of organogenesis (previous year *n* − 1. See details in **Supporting Information—Appendix 2**). For each architectural variable, a PLS regression was computed following the procedure of PLS in SIMCA software (Eriksson *et al.* 2006), which was translated in R to allow automatization of several variations of the analyses: linear PLS for ASL, NL and length of male flowers on the annual shoot, and logistic PLS (Bastien *et al.* 2005) for the number of male cones (black spruce), presence of male cones (pine species), number of branches (all in whorls for pines, whorled and interwhorl branches separately for black spruce), number of cones and polycyclism rate. We used *r*^2^ and *Q*^2^ statistics to evaluate the model, and the confidence interval of partial correlation coefficients to evaluate the significance of each variable. The *r*^2^ reflects the fit of the model to our data set, whereas the *Q*^2^ reflects the robustness of the model (Tenenhaus 1998). For each architectural parameter, we kept the best model according to *Q*^2^ with only significant variables. For a detailed description of the method, see **Supporting Information—Appendix 3**.

We finally focused on the variable importance in the projection (VIP), as described in Tenenhaus (1998). Variable importance in the projection is a coefficient >0, computed for each variable to reflect its relative weight in the model. As the sum of VIPs is always the total number of variables in the model, then variables with a VIP >1 are the most important for the prediction.

Variable importance in the projection absolute values must not be compared among different analyses nor among species. Variable importance in the projection is determined as a relative weight within the set of significant variables for a given analysis. The fact that some architectural traits show higher VIPs in response to climate variables than other traits does not mean that they are more responsive to climate: the real impact of any variable depends on both the number of significant variables and of the global correlation coefficient of the model (*r*^2^ and *Q*^2^).

When individual branches were significantly represented in the results of an analysis, with at least five top or medium and five low branches, we compared the VIPs mean or median of these classes with a *t*-test or a Mann–Whitney (Wilcoxon) test, respectively, depending on data distribution (normal or not).

## Results

### Mean architectural variables

Annual shoot length, the total number of branches and whorled branches were highly variable, and decreased with age and with increasing branching order for all species (Table 4). On black spruce, branching rate showed an abrupt drop from the trunk to the third branching order. It decreased more rapidly for interwhorl than for whorl branches: this species had about three times more interwhorl than whorled branches on vigorous axes (annual young shoots from order 1 and 2), interwhorl branches nearly disappearing on order 3, but not whorls. Needle length had a lower variability than branching rate among branching orders and with age, particularly in the case of black spruce, characterized by needles that did not shorten with axis age. Needle length also had by far the lowest variation coefficients (computed from mean and SD given in Table 4) within each branching order and age class (0.17 % ± 0.05), compared to all other architectural traits (from 0.56 % ± 0.22 for ASL to 1.65 % ± 0.90 for int ram). Male and female cones could be found together on the same annual shoot in black spruce but never in pine species. For black spruce, male cones were not found on the trunk, whereas female cones were absent at branching orders 2 and 3 for old axes. Female cones were quite rare for Eastern white pine. Polycyclism was found only on pines, systematically on Jack pine vigorous shoots, but more rarely on Eastern white pine (<2 % of annual shoots of the most polycyclic category).

**Table 4.** Descriptive statistics of studied architectural variables by species, axis hierarchical order and ontogenic age: mean, SD, range and percentage of occurrence. In Age column, the number in brackets is the number of observations. In the columns for ASL, needle length (NL), number of male cones for black spruce (N ♂), length of male cones on annual shoot for pines (Lg ♂), number of female cones (N ♀), number of branches in the pseudowhorl for black spruce and in the whorl for pines (whorl ram) and number of interwhorl branches for black spruce (int ram), the first row of each cell corresponds to the mean, the second row (in italic) to the SD, the third row, between square brackets, to the range of the variable (if SD > 0). For N ♂ and Lg ♂ for pines, a fourth row gives the percentage of twigs bearing male cones. Polycyclism column (polyc) reports for each number of cycles (1–3) the percentage of concerned axes.

Species	Order	Age	ASL	NL	N ♂	N ♀	whorl ram	int ram	Polyc
Black spruce	1	<45 *(7*)	210.4 *31.13* [179, 261]	7.86 *1.46* [6, 10]	0 *0*	0 *0*	6.57 *1.9* [4, 9]	16.29 *3.68* [11, 22]	1:100 %
		>45 (*26*)	127.1 *57.84* [48, 234]	5.77 *1.3* [4, 9]	0 *0*	0.17 *0.38* [0, 1]	4.54 *1.84* [2, 9]	9.42 *6.77* [1, 31]	1:100 %
	2	<35 (*817*)	39.62 *19.25* [5, 180]	7.89 *1.46* [4, 11]	0.08 *0.49* [0, 5]	0.05 *0.33* [0, 4]	3.48 *1.64* [0, 9]	1.31 *1.75* [0, 13]	1:100 %
		>35 (*270*)	31.64 *11.81* [4, 79]	8.4 *1.75* [4, 12]	0.03 *0.22* [0, 2]	0 *0*	2.88 *1.7* [0, 11]	0.72 *1.08* [0, 5]	1:100 %
	3	<10 (*549*)	25.92 *11.06* [3, 70]	7.76 *1.54* [4, 12]	0.31 *0.92* [0, 6]	0.05 *0.3* [0, 3]	1.94 *1.39* [0, 8]	0.18 *0.57* [0, 4]	1:100 %
		>10 (*209*)	25.4 *11.37* [5, 68]	7.92 *1.57* [3, 13]	0.06 *0.34* [0, 3]	0 *0*	1.61 *1.1* [0, 5]	0.25 *0.74* [0, 5]	1:100 %

Species	Order	Age	ASL	NL	L ♂	N ♀	whorl ram	int ram	Polyc
Jack pine	1	[62–75] (*13*)	114.3 *26.2* [82, 178]	28.92 *0.82* [28.33, 29.5]	0 *0*	3.69 *1.44* [1, 6]	7.46 *1.76* [5, 10]	NA	1:0 % 2:38.5 % 3:61.5 %
	2	<40 (*776*)	54.61 *34.96* [3, 193]	27.21 *5.57* [10, 39]	0.28 *1.66* [0, 16] 3.2 %	1.18 *1.24* [0, 6]	2.82 *1.94* [0, 8]	NA	1:35.5 % 2:64.4 % 3:0.1 %
		>40 (*176*)	30.57 *16.75* [4, 88]	23.24 *4.61* [9, 34]	1.64 *3.68* [0, 19] 21 %	0.56 *0.72* [0, 2]	1.42 *1.34* [0, 6]	NA	1:60.8 % 2:39.2 %
	3	<20 (*1008*)	22.49 *16.5* [2, 119]	23.93 *5.35* [6, 38]	1.05 *2.97* [0, 18] 14.1 %	0.14 *0.45* [0, 3]	0.65 *0.93* [0, 6]	NA	1:90.6 % 2:9.4 %
		>20 (*225*)	19.26 *12.35* [1, 70]	22.3 *3.84* [8, 31]	1.03 *2.62* [0, 15] 16.4 %	0.16 *0.43* [0, 2]	0.74 *0.86* [0, 4]	NA	1:87.11 % 2:12.89 %

Species	Order	Age	ASL	NL	L ♂	N ♀	whorl ram	int ram	Polyc
Eastern white pine	2	<30 (*639*)	109.9 *104.6* [4, 728]	79.03 *13.1* [52, 103]	0.72 *6* [0, 90] 2.03 %	0.05 [0, 3]	2 *2.09* [0, 13]	NA	1:98.1 % 2:1.9 %
		>30 (*503*)	80.57 *73.9* [6, 408]	76.12 *11.6* [44, 102]	0.92 *4.6* [0, 45] 5.2 %	0.01 *0.08* [0, 1]	1.44 *1.38* [0, 6]	NA	1:100 %
	3	<20 (*658*)	38.21 *32.8* [2, 452]	74.47 *12.11* [31, 101]	0.69 *3.9* [0, 85] 4.9 %	0.01 *0.55* [0, 4]	0.59 *0.85* [0, 7]	NA	1:99.9 % 2:0.1 %
		>20 (*547*)	23.6 *13.99* [4, 139]	71.18 *11.8* [37, 99]	1.34 *3.7* [0, 22] 12.8 %	0 *0*	0.27 *0.49* [0, 2]	NA	1:100 %

As we investigated many architectural variables for the three species, with several options for each model, we do not display all the detailed results. However, we present, as an example, the detailed results for one of these variables—ASL; details of all other analyses are presented in **Supporting Information—Appendix 4**. To fulfil our objectives, only the relative weight of predictors and the sign of the correlation are presented (Figs 3 and 4). Partial least square analyses were performed with all variables together (topology, ontogeny, climate and local variables), but synthetic results are presented in three parts for better comprehension: (i) topology and ontogeny (Fig. 3), to highlight the weight of tree-related factors on architectural development; (ii) climate (Fig. 4), to show the influence of interannual climate variability and the potential impact of climate change; and finally (iii) local variables **[see****Supporting Information—Appendix 4****]**, to demonstrate the influence of branch position in the crown.

### Annual shoot length

**Figure 2.**
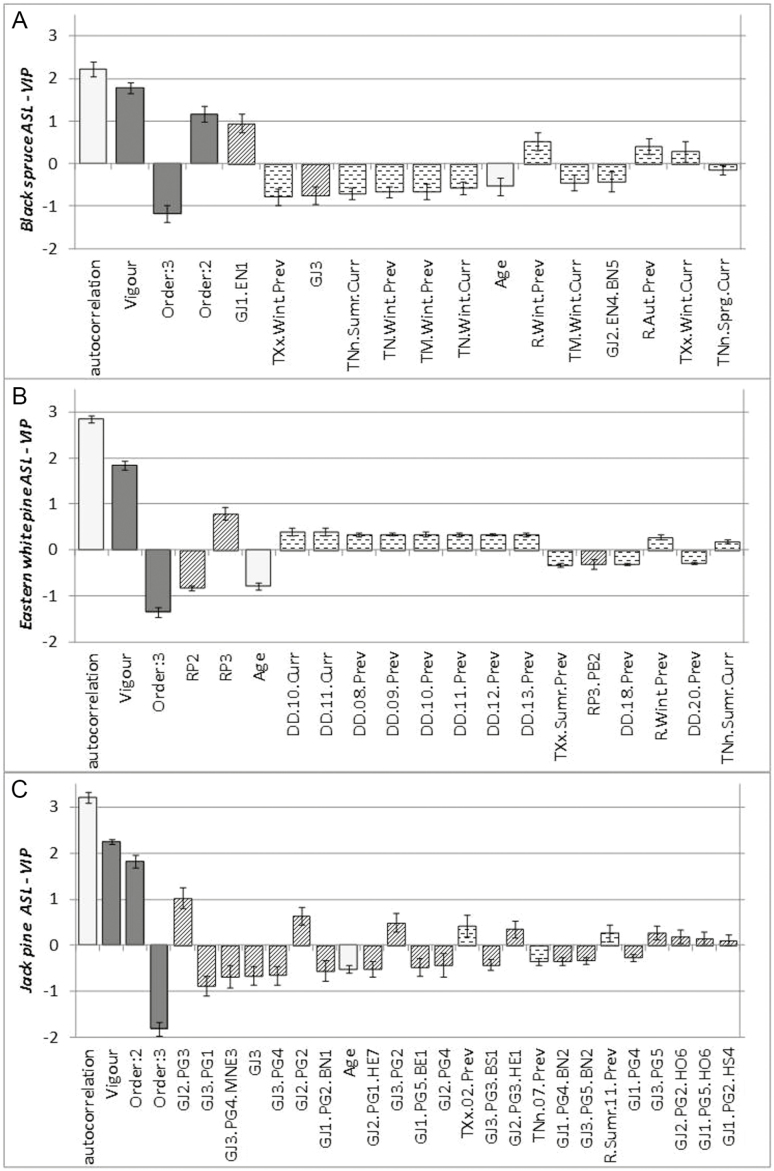
Partial correlation coefficients of variables in ASL PLS models. Letters (A), (B) and (C) plot for black spruce, Eastern white pine and Jack pine, respectively. Ontogenic variables appear in white, topologic variables in dark grey, local fixed effects in hatching and climatic variables in dashed lines. Variables are sorted by descending VIP from left to right. *R*^2^ are 0.633, 0.795 and 0.742 for black spruce, Eastern white pine and Jack pine, respectively; *Q*^2^ are 0.626, 0.794 and 0.736 in the same order. Local fixed effect may be related to: (i) site effect (GJ and site number for Grands Jardins, RP and site number for Réserve Papineau); (ii) tree effect (the site name, a dot and EN and tree number for black spruce, PB and tree number for Eastern white pine, PG and tree number for Jack pine) or (iii) branch effect (the tree name, a dot and a combination of two letters indicating the position within the crown, H-M-B for high, medium, low, the orientation = cardinal point N-E-S-W potentially combined by two, and the branch number). Climatic variables may correspond to organogenesis year (previous year, variable name ending by Prev) or elongating year (current year ending by Curr), and may be rainfall (begin with R.), absolute minimum temperature (begin with TNn.), mean minimum temperature (TN.), mean temperature (TM.), mean maximum temperature (TX.), absolute maximum temperature (TXx.) or degree-day (DD.). R, TNn, TN, TM, TX and TXx are calculated on a period of the year, indicated by the middle part of variable name, Wint for January–March, Sprg for April–June, Sumr for July–September, Aut for October–December, or a number corresponding to the month. Degree-days are calculated over the year, but with a basis corresponding to the number appearing in the middle of variable name.

Figure 2A–C plots partial correlation coefficients and VIPs of the PLS regression for ASL, for black spruce (*Q*^2^ = 62.6 %, *R*^2^ = 63.3 %), Eastern white pine (*Q*^2^ = 79.4 %, *R*^2^ = 79.5 %) and Jack pine (*Q*^2^ = 73.6 %, *R*^2^ = 74.2 %), respectively. Ontogenic and topological variables had the highest contribution to the model (VIP > 1). Other significant variables, including those related to climate, had less weight in the model. For the three species, the best predictors were the length of the previous annual shoot (autocorrelation), the relative vigour index and the branching order, ASL decreasing with increasing order. For Jack pine, many local factors (site, tree, branch) but only two or three climatic variables had a significant influence. Conversely, for Eastern white pine, only site was significant among local factors, while climate variables were more represented, although all weaker. Black spruce was the only species with VIPs above 0.75 for climatic variables; a negative correlation was noted for this species with previous-year winter absolute maximum temperature. For Eastern white pine, degree * days and temperature were dominant in the response to climate; a positive impact of moderate temperatures (neither too hot, nor too cold) of the current and previous years (DD 8–13, minimum temperature of current summer) was observed, as well as a negative impact of a very hot previous summer (maximum temperature and DD 18–20). The response of Jack pine to temperature was weak, with a positive influence of previous February absolute maximum temperature and a negative response to previous July absolute minimum temperature.

Because of the low number of significant local variables in ASL models, branch positions could only be compared statistically in these models for Jack pine, showing higher signed VIPs in top crown compared to low crown. For the other two species, low crown was consistently represented with only negative coefficients.

### Topological and ontogenic variables

**Figure 3.**
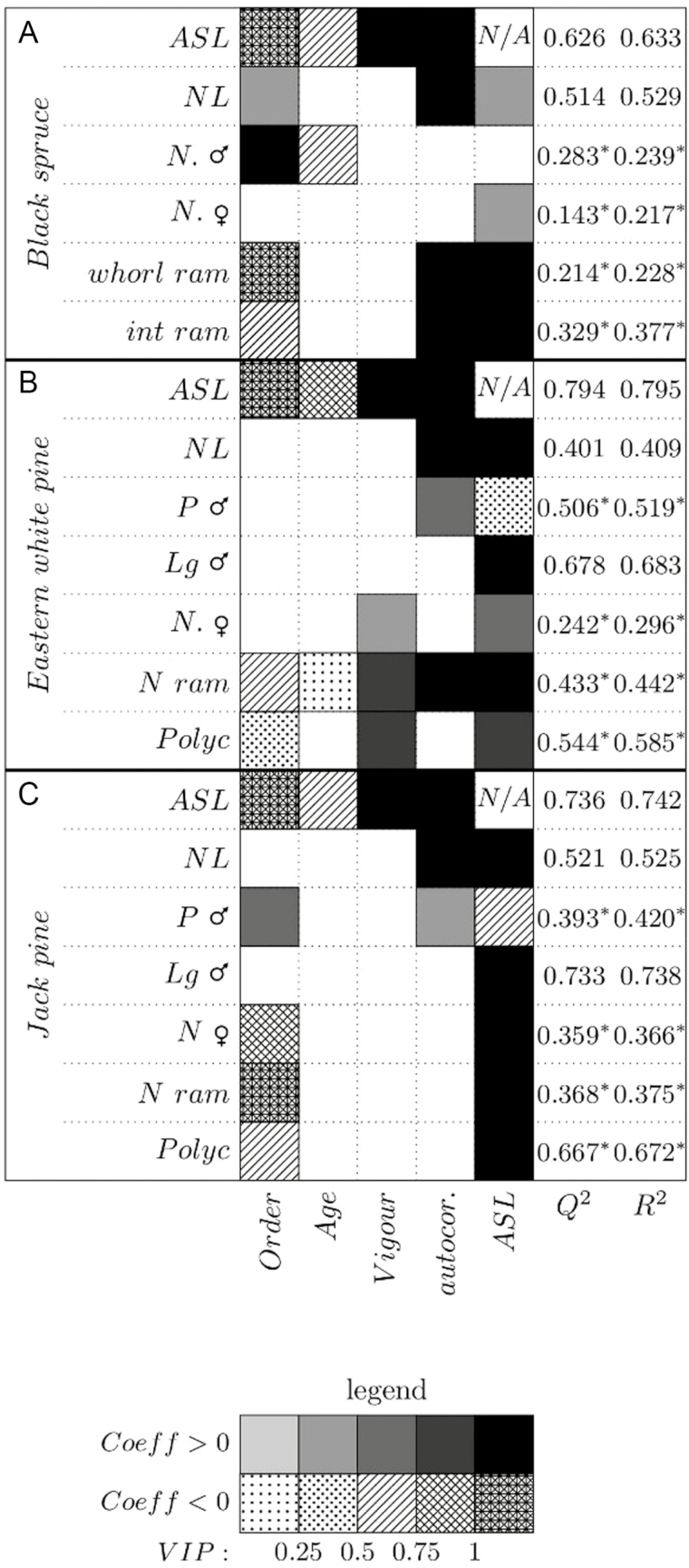
Dependant topological and ontogenic variables for all models, for black spruce (A), Eastern white pine (B) and Jack pine (C) vs. independent explanatory variables. Positive correlation appears in greyscale, whereas negative correlation appears with dots and hatchings. A star for *Q*^2^ and *R*^2^ indicates a logistic regression model, hence *R*^2^ and *Q*^2^ correspond to McFadden pseudo-*R*^2^ calculation method (McFadden 1973).

Figure 3A–C presents the VIPs and the sign of the partial correlation coefficients of topological and ontogenic variables for black spruce, Eastern white pine and Jack pine, respectively. For the three studied species, ASL had a dominant influence on architectural development. Except for the number of male cones of black spruce, it was significant for all variables, with a high VIP (>1) in >50% of the cases, particularly for branching and needle length. The probability of a branch to develop male cones was negatively correlated with ASL for both pine species. Conversely, within the short annual shoots bearing male cones, the length of the axis bearing these cones increased for both species with shoot length. All variables were negatively correlated with axis hierarchical order, except male cones, which were more frequent in high orders, and black spruce needles, that were shorter on the trunk than on branches. Branch relative vigour was less relevant than both ASL and branch hierarchical order, with no influence on architectural variables for either black spruce or Jack pine. It was, however, significant with a high VIP for polycyclism, fructification and branching in Eastern white pine. The autocorrelation (previous-year value) was important in ASL and needle length for all species, for male cones in pine species, and in the branching rate of black spruce and Eastern white pine. In contrast, autocorrelation had no weight in fructification of any species, nor in polycyclism; these variables showed sharp changes from a given year to the next within branches. Shoot length was negatively correlated with axes age for all species, demonstrating a clear impact of aging on absolute vigour of axes, despite the small VIP associated with age. Age also influenced negatively black spruce male cone number and Eastern white pine branching.

Branch position in the crown had a significant influence on architectural variables. The number of male cones, of interwhorl branches and of cones for black spruce, as well as the probability of male cones for Eastern white pine and the ASL of Jack pine, showed a higher VIP for top than for low branches (Annex 4, tables BS3, BS4, BS6, EWP2, JP1).

### Climatic variables

**Figure 4.**
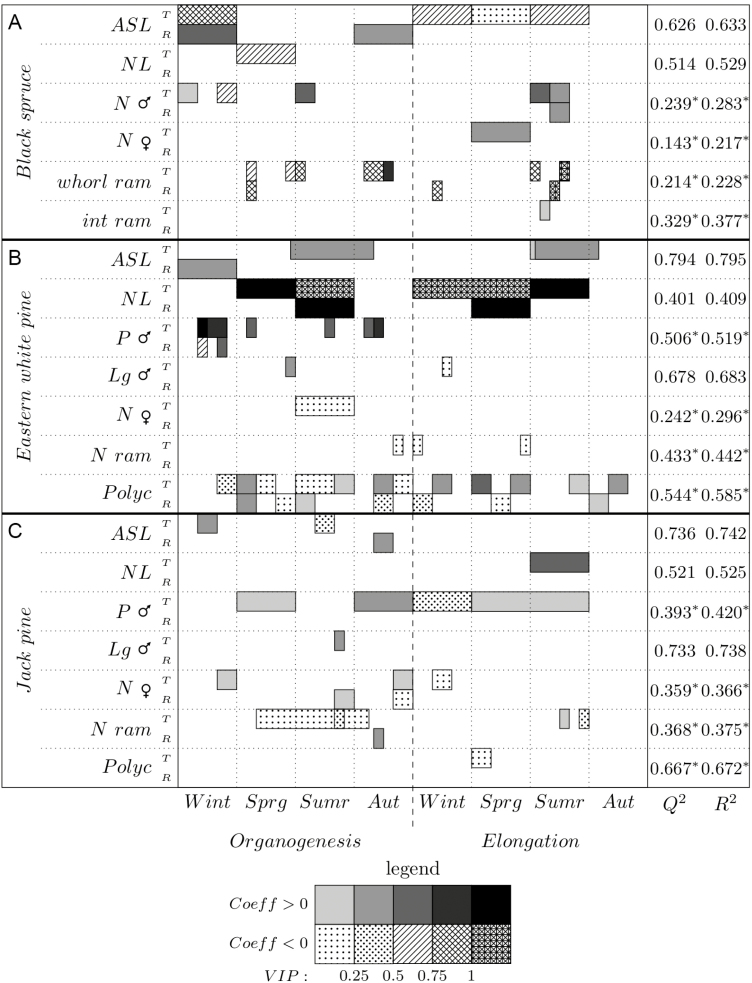
Climatic variables for each model, for black spruce (A), Eastern white pine (B) and Jack pine (C). Positive coefficients appear in grey scale, whereas negative ones appear with dots and hatchings. T stands for temperature, R for précipitation, Wint for winter (January–March), Sprg for spring (April–June), Sumr for summer (July–September) and Fall for October–December. The last columns on the right give the *Q*^2^ and *r*^2^ for each model.

Figure 4A–C shows the VIPs and the sign of the partial correlation coefficients of climate variables for black spruce, Eastern white pine and Jack pine, respectively.

Higher temperatures during elongation led to a decline in shoot length growth for black spruce and Jack pine, as well as a decline in branching and polycyclism for Jack pine, thus in crown development as a whole. Conversely, their reproduction (both male and female cones) was stimulated. However, for Eastern white pine, shoot length and male cone production were boosted by higher temperatures, while female cone production and branching rates were reduced. For this later species, polycyclism increased with higher temperatures from late summer of year *n* − 1 to autumn of year *n*, but declined with higher temperatures from the middle of spring to the middle of summer of year *n* − 1. For both pines, a hotter summer in year *n* increased needle length of year *n*. But Eastern white pine needle length was also strongly influenced by the temperature in spring *n* − 1 (positively), and in summer *n* − 1 and both winter and spring *n* (negatively). Surprisingly, black spruce needle length was negatively, but very weakly, related to temperatures of spring *n* − 1. Its interwhorl branching pattern was independent from climate.

For the three studied species in general, precipitation had less influence than temperature on architectural development and reproduction, with a few exceptions; ASL for the three species, and male flowering for Eastern white pine, were strongly favoured by higher precipitation. To a lesser extent, black spruce male flowering and Jack pine branching rate also benefited from a rainy summer *n* and autumn *n* − 1, respectively. Precipitation was negatively correlated with black spruce branching rate and had variable effects on Eastern white pine polycyclism.

## Discussion

### The strong weight of ontogeny and topology

Our results showed that the development of tree crown and reproduction were mainly controlled by ASL and by variables related to branch topology (branching order, relative vigour) and ontogeny (tree age, ontogenic shoot age, shoot length of year *n* − 1). The overall weight of climate interannual variability, when compared to these factors, was weaker and differed among the studied species. Chaubert-Pereira *et al.* (2009) already demonstrated changes in development patterns of trees with ontogenic phases, varying among species and individual trees within species, and modulating the growth response to climate.

Annual shoot length was the main driver of architectural development at the local scale. Axis vigour was consistently the strongest explanatory factor for architectural development in previous studies (Girard *et al.* 2011, 2012). For both pines and spruce, ASL was strongly correlated with branch position in the crown, thus with branch age, in accordance with Colombo *et al.* (2006). For young to adult conifers, ASL also decreased from the hierarchical order 1 (trunk) to the following orders 2 and 3 (main branches and twigs, respectively) (Millet 2012; Vennetier *et al.* 2013). As a consequence, shoot length could be considered as an integrating trait for branch intrinsic vigour, position and hierarchy. Functionally, shoot length also reflects physiological age of apical meristems (Barthélémy and Caraglio 2007), which reinforces the necessity to (i) compute this trait in architectural modelling and (ii) consider it in the set of explanatory variables when studying other architectural traits (e.g. branching rates, needle length and number, polycyclism, reproduction). Accordingly, the strong weight of autocorrelation for most variables was related to multiple interacting effects controlling the mean ASL independently from shoot hierarchical level and position: (i) site mean fertility for all trees of a given site; (ii) inter-tree variations (genetics, specific position in the site, tree history); (iii) at the branch scale, mean vigour status and age; (iv) the dependence of each annual shoot on the general conditions during bud morphogenesis, occurring in year *n* − 1 (Hover *et al.* 2017); and (v) the ability of trees to build up reserves to be used later, if the climate is favourable in autumn *n* − 1 (Körner 2003).

Independently from branch vigour, architectural variables can vary due to branch position in the crown, as already shown on both conifers (Thabeet *et al.* 2009) and broad-leaved species (Normand *et al.* 2009). Consistently, individual branch label **[see****Supporting Information—Appendix 4****]** influenced several variables for the three studied species, although shoot length and branch vigour were included in the analyses. However, the comparison of VIPs between branches from high and low crown in our model outputs was an indirect measure of the influence of branch position: branch position in the crown should be used directly as an additional independent topological variable in future investigations (Girard *et al.* 2011, 2012).

**Figure 5.**
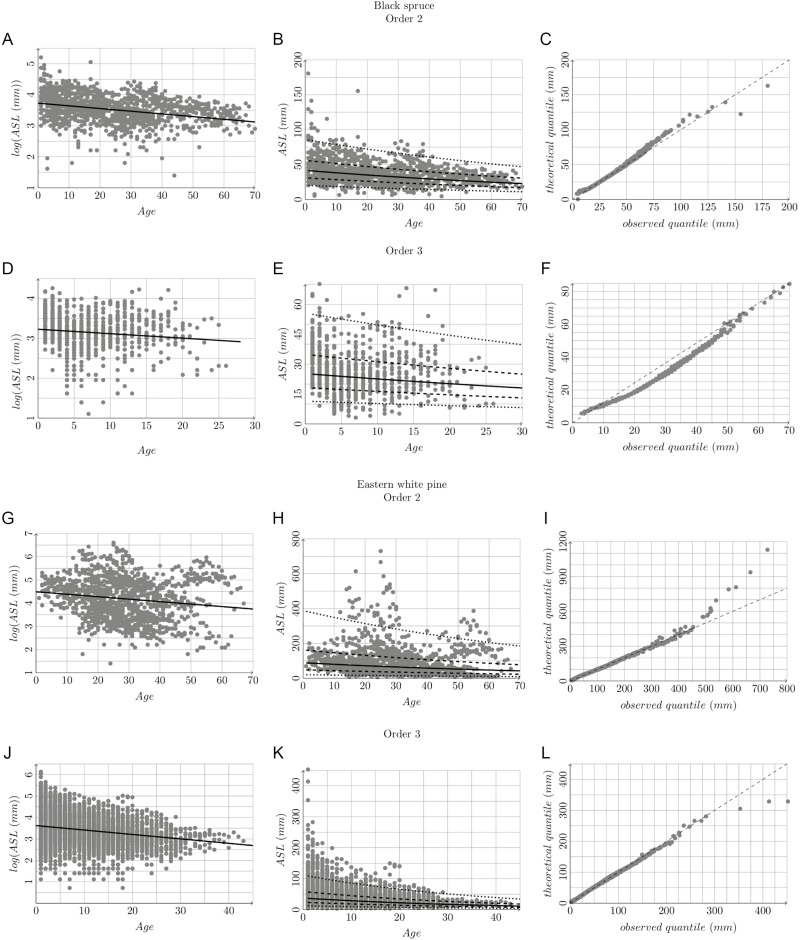

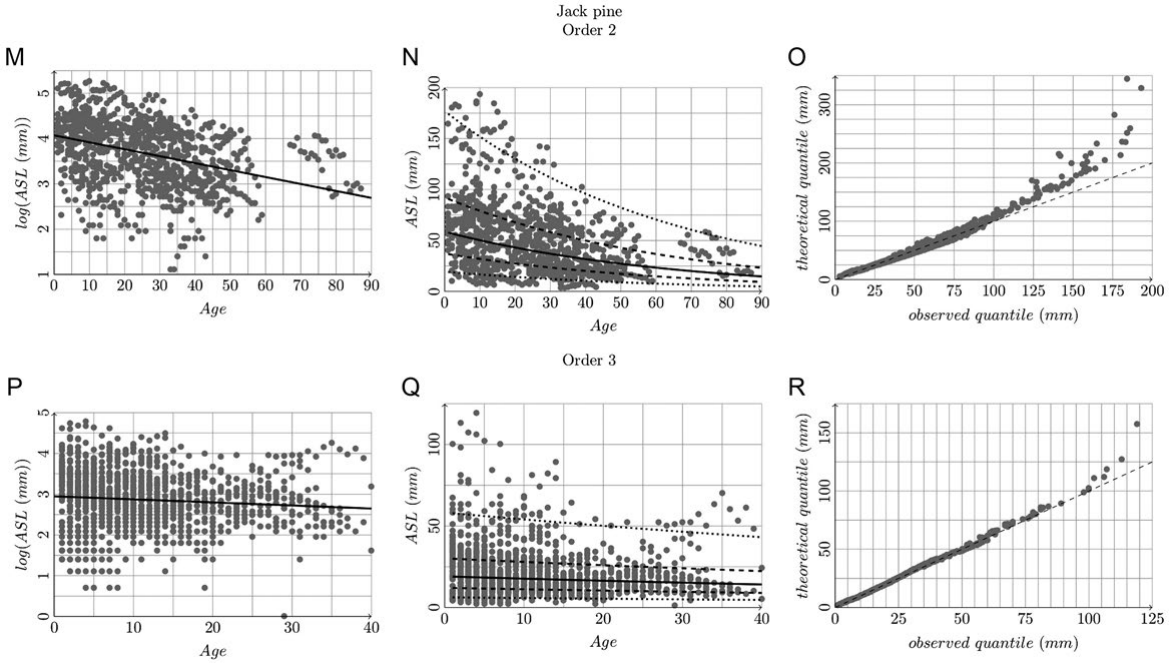
Relationship between log(ASL) or ASL and age, and related quantile–quantile plots, for order 2 and 3 branches of black spruce, Eastern white pine and Jack pine. For log(ASL), the line plots the linear model. On ASL distribution, the solid line plots the probability 0.5, the two dashed lines the probability 0.25 and 0.75, the two dotted lines the probability 0.05 and 0.95.

Branch hierarchy had a significant influence on most architectural variables, for all studied species (Fig. 3). However, axis relative vigour (Fig. 5) had no influence on architectural variables and reproduction of black spruce and Jack pine. For these latter two species, branching order was a strong driver (Colombo and Templeton 2006) (Fig. 3), lessening the relative weight of shoot length in trait variations within each order. Axis relative vigour was, however, significant for branching, polycyclism and cone production in Eastern white pine. This is due to larger variations of these variables and of axis vigour within each branching order (high relative SD; Table 4). Such variability lessens the influence of branching order for this species (Fig. 3B) and proportionally increases the weight of axes relative vigour. In this study, we sampled only relatively young adult trees, for which the freshly established structure and axis hierarchy is stable. The branching order should be considered with caution for older trees, for which loss of branch vitality with aging and branch mortality lead to their partial replacement by reiteration processes that break growth hierarchy between axes (Bégin and Filion 1999; Millet 2012). Although highly time-consuming, sampling orders 4 and higher would be useful for a more accurate description and modelling of the tree crown.

### Climate variability impacts crown development

The lower VIP for climate variables compared to ontogenic and topological variables does not suggest a low impact of climate on each branch, but instead a higher variability of architectural variables among sites, trees or branches than among years. This was the case, for example, for branching rates of most species, but also for ASL, which decreased sharply with both branch hierarchical order and age (from 2 to 5 times less from one order or age class to the other; Table 4). Comparatively, branching rate and ASL interannual variation along individual axes, due to climate, was limited to 20–50 % in this study. This response is consistent with previous studies (Girard *et al.* 2012; Feichtinger *et al.* 2015), and explains the low VIPs of climate variables in our results. Moreover, topological and ontogenic variables (position in the crown, hierarchical order, age of the tree, age of the branch and its relative vigour in its order) are fixed from the birth of the branch or evolve slowly and then have a fixed and monotonous effect with no or very low interannual variability. Conversely, climate leads to frequent interannual variations and to cumulative and additive effects, through long-lasting consequences of either a reduced branching rate or series of short needles or reduced needle number (Feichtinger *et al.* 2015); Vennetier *et al.* (2013) showed that a branching deficit, limiting potential leaf number, or reduced leaf number and size following repeated climate stresses, hold back tree leaf area. Such a reduction in leaf area, and thus light interception, may limit plant growth potential and vegetation productivity (Monteith 1977). Climate-related biotic attacks on weakened trees (Marini *et al.* 2012) also lengthen the recovery period.

The negative role of high temperatures from spring to autumn observed in shoot growth and architectural development of black spruce and Jack pine, and particularly the strong effect of maximum temperatures, was consistent with previous studies. Black spruce shows a reduction in the tree ring-width during or following hot years (Dang and Lieffers 1989; Nicault *et al.* 2014; Housset *et al.* 2015; Walker *et al.* 2015). This species seems more vulnerable to warming, and to the high water vapour deficit induced by warming (Van Herk *et al.* 2011), than to short droughts at normal temperature (Belien *et al.* 2012). This vulnerability is consistent throughout a large range of sites, including more fertile and cool sites (Walker and Johnstone 2014). Therefore, a large part of the contemporary distribution of black spruce, particularly at its southern edge, is predicted to be lost or threatened by 2060 (Joyce and Rehfeldt 2017). In addition, its radial growth may decrease at low latitudes, south of 47°N (Huang *et al.* 2010). For Jack pine, Wang *et al.* (2012) forecast an increase of productivity for a hotter climate with high precipitation, but a reduction in the case of reduced precipitation within the same time frame. This leads to potential interactions between climate and site conditions. On the shallow xeric soils of our studied sites, higher temperature may lead to water shortage in summer, which could explain the detrimental influence of maximum temperature of the growing season (Figs 2 and 4). A positive effect could exist on deep soils with a good water-holding capacity. Girardin *et al.* (2016) confirm a recent intensification of the impacts of hydroclimatic variability on the radial growth of both Jack pine and black spruce; these impacts seem mainly driven by a negative sensitivity to previous summer temperature and a positive sensitivity to summer soil moisture of both previous and current years. Guillemette *et al.* (2017) show that the annual risk of mortality in northern hardwoods and mixed woods of Quebec is 3.6 times lower where annual temperature is low (2.3 % at 0.8 °C) than in a location where annual temperature is high (8.1 % at 4 °C).

In parallel, we observed a positive correlation of tree crown development with winter temperatures and with minimum temperature in summer or spring, for the three studied species. This trend could be related to: (i) the detrimental impact of deep frost in winter, which can affect shoot terminal buds, and (ii) the need for sufficiently high minimum temperatures in the short growing season, but without extremes, in order to launch and sustain tree physiological activity. These thresholds of minimum temperatures are particularly critical in spring, for the activation of photosynthesis and respiration (Rayment and Jarvis 1999). They are also important in autumn to sustain root growth (De Barba *et al.* 2016), allow late polycyclism (Fig. 4B) and to build up carbohydrate reserves when photosynthesis remains active, while C sink demand for primary growth is limited (Körner 2003). Consistently and for the same reasons, very low temperatures in winter contribute to higher tree mortality rates during the following year in Europe, particularly in its northern regions (Neumann *et al.* 2017).

Unlike Jack pine and black spruce, Eastern white pine was not negatively affected by high temperatures. (Girardin *et al.* 2016) show that its radial growth is not influenced by temperature in any season and we reported that shoot length growth was even favoured by higher temperature in our sites (Fig. 4B). The positive correlation between polycyclism and high temperature of the current growing season, and the negative correlation between polycyclism and high temperatures of the past year (Fig. 4B), may not be contradictory but may rather show an interdependency. Indeed, two growth units can be prepared in the terminal bud of elongating shoots, as observed for other polycyclic species (Hover *et al.* 2017). If temperatures are high enough during of a given growing season, the first of these units can develop as a second cycle for the current year, and only the second unit remains thus available at the beginning of the next growing season. If the first unit does not develop, two growth units are available for next year, increasing the probability of polycyclism, even without high temperatures. Moreover, as demonstrated by Buissart *et al.* (2015) through shoot pith diameter, organogenesis is competing for resources with primary growth during year *n* − 1. Therefore, climate conditions enabling a prolonged vigorous growth in the autumn of any given year may lead to fewer potential internodes prepared in the bud for the following year.

The three studied species followed the normal patterns of conifers for reproduction (Figs 3 and 4); male cones were more abundant or frequent in high branching orders (here mainly order 3) and on the weak axis (Caraglio *et al.* 2007; Thabeet *et al.* 2009; Ne’eman *et al.* 2011), while female cones were mainly limited to the trunk or main branches (order 2) and to the most vigorous axes. Consistently, high temperatures which weaken trees and limit shoot growth also reduced female cone numbers and increase investment in pollen production (Fig. 4). The lack of influence of autocorrelation with fructification, whatever the species, shows that despite the weight of branch position, vigour and hierarchy in reproduction patterns, interannual climate variability and the intrinsic irregularity of tree reproduction (Pearse *et al.* 2017) constrain cone production.

Compared to temperature, the rainfall variability had little influence on the shoot growth and architectural development of the three studied species. As only one drought was recorded in the last 15 years in the study areas, this lack of statistical responsiveness of tree development to precipitation patterns was not surprising. However, we observed evidence of the potential role of precipitation in a changing climate; higher precipitation in autumn *n* − 1 or winter (the periods and their length differ among species) improved shoot length of the three species and branching rate for Jack pine (Fig. 4). As most of the precipitation in late autumn and winter occurs as snow, it can be interpreted as (i) a protection of the soil against extreme frost, and (ii) a long-lasting water reserve released in spring which could be reduced in the future (Creed *et al.* 2015), inducing water stress for the growing season.

Globally, the response of the studied species to climate, and particularly the negative role of high temperature in the case of Jack pine and black spruce, confirmed that the theoretical improvement of tree growth and forest productivity with global change (lengthening of the growing season and elevated atmospheric CO_2_) could be offset by many other factors such as genetics, responsiveness to the photoperiod or vernalization processes. These important factors of tree plasticity and acclimation are set to optimize tree growth and survival with present and recent climate and site conditions (Clark *et al.* 2014; Creed *et al.* 2015). Any change that unbalances these relationships, and particularly any increase in drought stress, may put trees at risk (Choat *et al.* 2012), even if drought occurs for only a short period in summer, in cold-limited Canadian forests (Walker *et al.* 2015). However, Eastern white pine seems able to increase growth in response to slightly higher temperatures, provided that drought stress does not increase concurrently. Both Jack pine and black spruce bear semi-serotinous cones that constitute an aerial seed bank released under the heat of crown fires or after desiccation (Schooley *et al.* 1979; Pinard 1999). The increase in cone production with higher temperatures (Fig. 4A and C) may help these species to regenerate after episodes of forest dieback due directly to drought stress, or to a rise in wildfire frequency or biotic disturbances associated with such droughts. For Eastern white pine, the trend to limited cone production with higher temperatures in summer *n* − 1 (Fig. 4B) may be compensated by the opposite trend in shoot length growth and branch vigour that may improve in a slightly warmer climate, sustaining cones production. Due to the limited number of cones observed on studied Eastern white pine trees, this last result should be considered with prudence.

## Conclusions

Endogenous factors (ontogeny and topology) appeared more important drivers of adult tree architectural development than climate variability (exogenous factor) at branch and twig level. A similar study is required for other ontogenic stages, especially young trees in the establishment phase, to better understand the behaviour and survival of tree species in a context of global climate change.

Most of the architectural and growth traits studied depended on both the previous year (organogenesis) and present growing season (organ growth). This suggests that extreme climate events have at least a 2-year-long impact on tree development, as most of the traits are interdependent. In fact, these events probably have far longer effects, as suggested by the significant weight of autocorrelation that may prolong the immediate consequences.

Although dominated by endogenous factors, the architectural development of tree crown as a whole was also clearly dependent on interannual climate variability for studied species (Fig. 4). But the detailed climate signals may have been partly hidden by the analyses which pooled all branches. The responses to climate obtained were the strongest ones impacting consistently the whole crown (top to base, all hierarchical orders and all vigour classes), while architectural traits were highly dependent on topological factors. Additional analyses comparing and targeting separately branch position, hierarchy and vigour (Girard *et al.* 2011, 2012) are necessary for a more detailed assessment of tree architectural response to climate.

Another important step forward to accurately predict the leaf area from architectural analyses (and hence more accurately predict forest productivity and carbon storage) is to model the number of needles per annual shoot. Tree development also depends on needle and branch mortality; needle and branch life span are related to species development strategies, interacting with the effects of age, environmental conditions, competition and biotic interferences. These latter are prone to increase with climate change.

## Sources of Funding

This work was supported by funds from the France-Quebec Council for University Cooperation (CFQCU, grant number 2014-FQ-174602), the National Research Institute for Science and Technology for Environment and Agriculture (Irstea) and the Provence-Alpes-Côte d’Azur region (50 % PhD grant).

## Contributions by the Authors

Research design: M.V., F.G., S.D. and A.D.M. Field work and measures: F.B., M.V., F.G., S.D. and A.D.M. Statistical analyses: F.B. and M.V. Manuscript design, writing and proofing: M.V., F.B., F.G., S.D., A.D.M., Y.C., S.-A.S. and E.-A.N.

## Supplementary Material

Appendices

## Supporting Information

The following additional information is available in the online version of this article—

**Appendix 1.** Method to compute the branch relative vigour index.

**Appendix 2.** Climatic variables.

**Appendix 3.** Partial least square (PLS) analysis protocol.

**Appendix 4.** Parameters for all partial least square (PLS) models.
