# Supplementary material for: The relative weight of ontogeny, topology and climate in the architectural development of three North American conifers
Source: AoB Plants. 2018 Jul 31;10(4):ply045. doi: 10.1093/aobpla/ply045 (PMC6101484; doi:10.1093/aobpla/ply045)
Supplement: Appendices [file ply045_suppl_appendices.pdf]

Appendices

Appendix 1 – Method to compute the branch relative vigour index

For each species and for each branching order, log(ASL) was linearly correlated with branch Age (Fig. 5). Table a1 displays the parameters of linear models. Then we computed for each AS an index for its length, considering its position in a normal distribution with the mean dependant on the age and given by the linear regression, and the standard deviation (SD) corresponding to the standard deviation of the residuals of the linear model. For example, the indices of annual shoots which length are equal to the mean minus 2*SD, the mean and the mean plus 2*SD are respectively 0.05, 0.5 and 0.95. The vigour index of an axis is computed as the average of the indices of all its ASs. Thus the most vigorous the axis relatively to its branching order, the highest its mean index.

Tab a1 Parameters of linear models for log(ASL) vs Age fitted by species and branching order. Sd resid Column reports the standard deviation of the residuals. The p-value (P) for intercept (Int) and Age coefficient (coef) was always below $1*10^{-3}$.

Species	Order	int	coef	r^2	Sdresid.
Black spruce	2	3.73	$-8.67\ 10^{-3}$	0.960	0.440
	3	3.23	$-1.12\ 10^{-2}$	0.156	0.480
Eastern white pine	2	4.49	$-1.06\ 10^{-2}$	0.232	0.890
	3	3.62	$-2.62\ 10^{-2}$	0.777	0.665
Jack pine	2	4.07	$-1.53\ 10^{-2}$	0.125	0.672
	3	2.95	$-7.51\ 10^{-3}$	0.0941	0.678

Appendix 2 – Climatic variables

We used 1999-2013 daily data (minimum temperature, mean temperature, maximal temperature and total rainfall) from L'Etape weather station for Parc des grands jardins and 1999-2013 daily data (same variables) from Chénéville weather station for Réserve Papineau.
Environmental variables should have an impact on architecture, our hypothesis is that the environmental conditions occurring during organogenesis and elongation will shape the architecture. For studied species organogenesis occurs the year before the elongation. Thus, we calculated for each year between 2000 and 2013 the following variables (appear in bold the variable aliases):

- **TNn**: absolute minimum temperature in °C
- **TN**: minimum temperature (mean of minimum temperatures) in °C
- **TM**: mean temperature (mean of mean temperatures) in °C
- **TX**: maximum temperature (mean of maximum temperatures) in °C
- **TXx**: absolute maximum temperature in °C
- **R**: total rainfall in mm
- **DD**: degree-day in °C

These variables were computed for three time spans: seasonally(**Wint** for January to march, **Sprg** for April to June, **Sumr** for July to September and **Aut** for October to December), monthly (**01** for January, **02** for February, etc.) and a bimonthly (**01a** for the first half of January, **01b** for the second half of January, **02a** for the first half of February, etc.), the current year of elongation (year n = **Curr**) and the previous year (year n-1 = **Prev**). Variable aliases are then composed of three parts: climatic parameter first, then after a dot the period of the year and finally the year. **TM.06.Curr** stands for the mean temperature in June of the year n and **R.Sprg.Prev** stands for the total rainfall between April and June of year n-1. We also calculated **DD.x** as the degree-day with the basis x ($x\ integer\ \in [0, 20]$) in °C, for the actual and the previous year. Then **DD.10.Curr** stands for the degree-day with basis 10°C for the year n, whereas **DD.01.Prev** stands for the degree-day with basis 1°C for the year n-1.

Appendix 3 – PLS analysis protocol

The PLS algorithm operated as follow:

- For each architectural variable we performed six analyses: for all analyses we used as predictors the topological variables, autocorrelation (value of the previous annual shoot), and annual shoot length (except for ASL itself), then we added in turn seasonal climatic variables (with or without degree-day), monthly climatic variables (with or without degree-day) and bimonthly climatic variables (with or without degree-day).
- We started each analysis by a PLS regression with all variables, with 6 axis. We performed a linear PLS regression for log(ASL) log(NL) and Lg♂ (the last one only for twigs with male cones), and a logistic regression for P♂ (binomial), N ram, N cone, Polyc, int ram, and whorl ram. Each analysis then iterates the following steps:
    - For each axis, if all variables are significant, the model is accepted,
    - For each axis, if some variables are non-significant, they are removed and PLS regression is performed again
- At the end, the model with the highest Q^2 is selected.

Appendix 4 – Parameters for all PLS models

Tables a4-BS, -EWP and -JP give the significant variables of PLS models for black spruce, Jack pine and Eastern white pine respectively. ASL, NL and Lg male models correspond to linear PLS regression, whereas N male, int ram, whorl ram, N ram, N cone and Polyc correspond to logistic PLS regression. For logistic regression, each table first displays the classes used (lower | upper limit). Ontogenic (Ont, in white lines), topological (Topo, light grey italic) and climate (clim, darker grey bold) variables are sorted together by decreasing VIP absolute value, thus by relative weight in the model. Local variables (loc, in white lines) are sorted separately below the previous ones, by decreasing VIP as well.

The relative role of top and low crown branches (or middle and low branches for Eastern white pine) was analysed separately for relevant PLS models, those with at least 6 branches showing a significant weight in the model: number of male cones, inter-whorl branching and number of cone by AS for black spruce, probability of male cones, number of cones per AS and polycyclism for Eastern white pine, annual shoot length for Jack pine. The corresponding table is added on the right of the main VIP table, along with the result of the comparison, displaying the method (variance analysis or Wilcoxon test) and the probability of the difference. Branches are sorted by signed VIP (the sign of variable coefficient was added to its VIP) highlighting the relative role of branch position in the crown in these models (top = blue lines in bold, low = orange lines).

Tab a4-BS1: Annual shoot length (ASL) for black spruce

	Variable	Coef	VIP
Ont	Autocor	0.249	2.220
Topo	*Vigour*	*0.183*	*1.790*
Topo	*Order:3*	*-0.134*	*1.180*
Topo	*Order:2*	*0.133*	*1.170*
**Clim**	**TXx.Wint.Prev**	**-0.099**	**0.773**
**Clim**	**TNn.Sumr.Curr**	**-0.088**	**0.695**
**Clim**	**TN.Wint.Prev**	**-0.046**	**0.659**
**Clim**	**TM.Wint.Prev**	**-0.049**	**0.654**
**Clim**	**TN.Wint.Curr**	**-0.055**	**0.572**
Ont	Age	-0.102	0.529
**Clim**	**R.Wint.Prev**	**0.091**	**0.522**
**Clim**	**TM.Wint.Curr**	**-0.048**	**0.448**
**Clim**	**R.Aut.Prev**	**0.055**	**0.408**
**Clim**	**TXx.Wint.Curr**	**0.063**	**0.287**
**Clim**	**TNn.Sprg.Curr**	**-0.026**	**0.142**
Loc	GJ1.EN1	0.090	0.942
Loc	GJ3	-0.142	0.748
Loc	GJ2.EN4.BN5	-0.060	0.431

Tab a4-BS2: Needle length (NL) for black spruce

	Variable	Coef	VIP
Ont	autocorrelation	0.307	2.200
**Clim**	**TN.04.Prev**	**-0.076**	**0.503**
**Clim**	**TXx.4.Prev**	**-0.052**	**0.500**
Topo	*Order:1*	*-0.084*	*0.487*
Ont	ASL	0.104	0.468
Loc	GJ3.EN1	0.158	1.630
Loc	GJ2.EN5	0.149	1.330
Loc	GJ2.EN2	-0.151	1.110
Loc	GJ2.EN3.BS6	-0.093	1.010
Loc	GJ1.EN3	-0.145	0.783
Loc	GJ1.EN1.HS5	-0.053	0.526
Loc	GJ1.EN4	-0.071	0.492
Loc	GJ1.EN2.HO5	0.058	0.465
Loc	GJ3.EN4	-0.093	0.411

Tab a4-BS3: Number of male cones per AS (N male) for black spruce

**Classes span**
0|≤1
1|≤2
2|≤3
3|≤4
4|≤5
5|6

	**Variable**	**Coef**	**VIP**
Topo	*Order:1*	*-1.620*	*1.180*
Ont	Age	-0.594	0.683
**Clim**	**TNn.07.Prev**	**0.514**	**0.615**
**Clim**	**TNn.07.Curr**	**0.246**	**0.565**
**Clim**	**TXx.03.Prev**	**-0.445**	**0.525**
**Clim**	**TNn.03.Prev**	**-0.443**	**0.521**
**Clim**	**TN.03.Prev**	**-0.264**	**0.397**
**Clim**	**R.08.Curr**	**0.287**	**0.389**
Topo	*Order:3*	*0.190*	*0.385*
**Clim**	**TNn.08.Curr**	**0.231**	**0.344**
**Clim**	**TN.01.Prev**	**0.115**	**0.215**
Loc	GJ3.EN4	-3.570	2.750
Loc	GJ1.EN4	-3.280	2.390
Loc	GJ1.EN3	-2.560	1.820
Loc	GJ2.EN5.BS1	-2.020	1.290
Loc	GJ1.EN1.BN2	-1.890	1.250
Loc	GJ1.EN2.BE2	-1.860	1.230
Loc	GJ1.EN4.BS2	-1.050	1.200
Loc	GJ2.EN3.BS5	-1.770	1.110
Loc	GJ3.EN3.BS2	-1.520	1.040
Loc	GJ3.EN4.HN5	-0.762	1.010
Loc	GJ1.EN5.BN5	-1.370	0.932
Loc	GJ1.EN4.HS5	-0.789	0.930
Loc	GJ1.EN1.BS1	-1.310	0.846
Loc	GJ1.EN2.HO5	-1.320	0.827
Loc	GJ3.EN4.BS1	-0.676	0.818
Loc	GJ2	0.189	0.325
Loc	GJ2.EN4.HN2	0.265	0.282
Loc	GJ2.EN4	0.119	0.226
Loc	GJ1.EN5.HS4	0.216	0.181
Loc	GJ2.EN1	0.063	0.173
Loc	GJ2.EN1.BO2	0.065	0.154
Loc	GJ3.EN1.HN6	0.131	0.129
Loc	GJ1.EN1.HS5	0.102	0.104

Top vs low branches

	**Variable**	**+/- VIP**
**top**	**GJ2.EN4.HN2**	**0.282**
**top**	**GJ1.EN5.HS4**	**0.181**
low	GJ2.EN1.BO2	0.154
**top**	**GJ3.EN1.HN6**	**0.129**
**top**	**GJ1.EN1.HS5**	**0.104**
low	GJ3.EN4.BS1	-0.818
**top**	**GJ1.EN2.HO5**	**-0.827**
low	GJ1.EN1.BS1	-0.846
**top**	**GJ1.EN4.HS5**	**-0.930**
low	GJ1.EN5.BN5	-0.932
**top**	**GJ3.EN4.HN5**	**-1.010**
low	GJ3.EN3.BS2	-1.040
low	GJ2.EN3.BS5	-1.110
low	GJ1.EN4.BS2	-1.200
low	GJ1.EN2.BE2	-1.230
low	GJ1.EN1.BN2	-1.250
low	GJ2.EN5.BS1	-1.290

Median VIP top: 0.104
Median VIP low: -1.075
Mann-Whitney (Wilcoxon) test
Mean rank top: 12.4286
Mean rank low : 6.6
W = 11.0 P = 0.0218

Top branches had a significantly higher signed VIP than low branches.

Tab a4-BS4: Inter-whorl branching for black spruce

Classes span	Classes span	Coef
0|≤1		
1|≤2	10|11	-11.900
2|≤3	11|12	-12.200
3|≤4	12|13	-12.500
4|≤5	13|15	-13.400
5|≤6	15|17	-14.000
6|≤7	17|19	-14.500
7|≤8	19|21	-15.100
8|≤9	21|22	-15.500
9|10	22|31	-16.200

	Variable	Coef	VIP
Ont	ASL	2.540	2.150
Ont	Autocor	0.598	2.030
Topo	*Order:3*	*-0.686*	*0.538*
Topo	*Order:1*	*0.494*	*0.367*
**Clim**	**DD.18.Curr**	**0.119**	**0.112**
**Clim**	**DD.19.Curr**	**0.131**	**0.103**
**Clim**	**DD.20.Curr**	**0.129**	**0.086**
Loc	GJ1.EN4.BS2	-1.900	1.380
Loc	GJ1.EN2.BE2	-1.850	1.290
Loc	GJ3.EN1.BN2	-1.830	1.280
Loc	GJ3.EN2.BN1	-1.900	1.190
Loc	GJ2.EN4.HN2	-1.580	1.110
Loc	GJ3.EN4.HN5	-1.460	1.000
Loc	GJ3.EN4.BS1	-1.260	0.855
Loc	GJ1.EN5.BN5	-1.280	0.849
Loc	GJ1.EN5.HS4	-1.160	0.846
Loc	GJ3.EN1.HN6	-1.210	0.843
Loc	GJ3.EN2.HS6	-1.130	0.676
Loc	GJ2.EN5.HS5	-0.895	0.663
Loc	GJ3.EN2	-0.573	0.348
Loc	GJ2.EN1	0.241	0.193
Loc	GJ3.EN1	-0.168	0.100

Top vs low branches

	Variable	+/-VIP
**top**	**GJ2.EN5.HS5**	**-0.663**
**top**	**GJ3.EN2.HS6**	**-0.676**
**top**	**GJ3.EN1.HN6**	**-0.843**
**top**	**GJ1.EN5.HS4**	**-0.846**
low	GJ1.EN5.BN5	-0.849
low	GJ3.EN4.BS1	-0.855
**top**	**GJ3.EN4.HN5**	**-1.000**
**top**	**GJ2.EN4.HN2**	**-1.110**
low	GJ3.EN2.BN1	-1.190
low	GJ3.EN1.BN2	-1.280
low	GJ1.EN2.BE2	-1.290
low	GJ1.EN4.BS2	-1.380

Variance analysis
t = 2.39375   P = 0.0377

Top branches had a significantly higher signed VIP than low branches.

Tab a4-BS5: Whorl branching for black spruce

Classes span
0|≤1
1|≤2
2|≤3
3|≤4
4|≤5
5|≤6
6|≤7
7|≤8
8|≤9
9|11

	Variable	Coef	VIP
Ont	Autocor	0.852	2.670
Ont	ASL	0.578	1.980
Topo	*Order:3*	*-0.215*	*1.680*
Topo	*Order:2*	*0.122*	*1.510*
**Clim**	**R.08a.Curr**	**-0.229**	**1.270**
**Clim**	**TNn.08b.Curr**	**-0.179**	**1.030**
**Clim**	**TX.07a.Curr**	**-0.126**	**0.990**
**Clim**	**TN.10b.Prev**	**-0.112**	**0.914**
**Clim**	**TNn.07a.Curr**	**-0.154**	**0.910**
**Clim**	**R.04b.Prev**	**-0.142**	**0.831**
**Clim**	**R.02a.Curr**	**0.171**	**0.830**
**Clim**	**TN.11b.Prev**	**0.203**	**0.800**
**Clim**	**TX.11a.Prev**	**-0.165**	**0.753**
**Clim**	**TN.06b.Prev**	**-0.125**	**0.664**
**Clim**	**TXx.04b.Curr**	**-0.117**	**0.585**
**Clim**	**TM.11a.Prev**	**-0.112**	**0.575**
Topo	*Order:1*	*0.265*	*0.518*
Loc	GJ3.EN2.BN1	-0.206	0.620
Loc	GJ1.EN5.BN5	-0.120	0.510
Loc	GJ3.EN4.BS1	-0.106	0.417
Loc	GJ2.EN4.HN2	-0.073	0.393
Loc	GJ1.EN5	-0.131	0.391
Loc	GJ2.EN1.HE5	-0.107	0.382
Loc	GJ2.EN4.BN5	-0.084	0.325
Loc	GJ3.EN1	0.126	0.308
Loc	GJ1	-0.108	0.239
Loc	GJ2.EN1	0.101	0.219

Tab a4-BS6: Number of cones by AS for black spruce

Classes span
0|≤1
1|≤2
2|≤3
3|4

	Variable	Coef	VIP
Ont	ASL	0.816	0.408
**Clim**	**TN.Sprg.Curr**	**0.523**	**0.258**
Loc	GJ3.EN2	-4.370	2.370
Loc	GJ3.EN1	-4.260	2.280
Loc	GJ3.EN2.BN1	-1.370	1.290
Loc	GJ2.EN5.BS1	-2.190	1.190
Loc	GJ3.EN5.BN1	-2.190	1.170
Loc	GJ1.EN1.BN2	-2.170	1.160
Loc	GJ3.EN1.BN2	-1.250	1.160
Loc	GJ1.EN2.BE2	-2.090	1.140
Loc	GJ1.EN4.BS2	-2.110	1.140
Loc	GJ2.EN4.BN5	-2.030	1.110
Loc	GJ1.EN2.BN1	-1.990	1.070
Loc	GJ2.EN3.BS5	-1.890	1.020
Loc	GJ3.EN3.HN6	-1.890	1.020
Loc	GJ2.EN1.BO2	-1.880	0.939
Loc	GJ3.EN4.HN5	-1.700	0.898
Loc	GJ2.EN2.Hx5	-1.610	0.844
Loc	GJ1.EN5.BN5	-1.460	0.802
Loc	GJ3.EN2.HS6	-0.782	0.799
Loc	GJ1.EN1.BS1	-1.470	0.784
Loc	GJ3.EN4.BS1	-1.430	0.782
Loc	GJ3.EN1.HN6	-0.780	0.773
Loc	GJ3.EN5.HN6	-1.430	0.763
Loc	GJ1.EN5.HS4	-1.290	0.696
Loc	GJ1.EN1.HS5	-1.220	0.649
Loc	GJ2.EN3.HS3	-1.120	0.597
Loc	GJ2.EN5.HS5	-1.010	0.527
Loc	GJ2.EN1	0.244	0.253
Loc	GJ2.EN1.HE5	0.267	0.191
Loc	GJ1.EN4.HS5	0.286	0.149
Loc	GJ1.EN2.HO5	0.281	0.144
Loc	GJ3.EN5	0.210	0.080

Top branches had a significantly higher signed VIP than low branches

Top vs low branches

	Variable	+/-VIP
**top**	**GJ2.EN1.HE5**	**0.191**
**top**	**GJ1.EN4.HS5**	**0.149**
**top**	**GJ1.EN2.HO5**	**0.144**
**top**	**GJ2.EN5.HS5**	**-0.527**
**top**	**GJ2.EN3.HS3**	**-0.597**
**top**	**GJ1.EN1.HS5**	**-0.649**
**top**	**GJ1.EN5.HS4**	**-0.696**
**top**	**GJ3.EN5.HN6**	**-0.763**
**top**	**GJ3.EN1.HN6**	**-0.773**
low	GJ3.EN4.BS1	-0.782
low	GJ1.EN1.BS1	-0.784
**top**	**GJ3.EN2.HS6**	**-0.799**
**top**	**GJ1.EN5.BN5**	**-0.802**
**top**	**GJ2.EN2.Hx5**	**-0.844**
**top**	**GJ3.EN4.HN5**	**-0.898**
low	GJ2.EN1.BO2	-0.939
low	GJ2.EN3.BS5	-1.020
**top**	**GJ3.EN3.HN6**	**-1.020**
low	GJ1.EN2.BN1	-1.070
low	GJ2.EN4.BN5	-1.110
low	GJ1.EN2.BE2	-1.140
low	GJ1.EN4.BS2	-1.140
low	GJ1.EN1.BN2	-1.160
low	GJ3.EN1.BN2	-1.160
low	GJ3.EN5.BN1	-1.170
low	GJ2.EN5.BS1	-1.190
low	GJ3.EN2.BN1	-1.290

Median VIP top: -0.7295
Median VIP low: -1.14
Mann-Whitney (Wilcoxon) test
Mean rank top: 8.357
Mean rank low : 20.077
W = 11.5   P = 0.0001

Tab a4-EWP1: Annual shoot length (ASL) for Eastern white pine

	**Variable**	**Coef**	**VIP**
Ont	Autocor	0.513	2.850
Topo	*Vigour*	*0.142*	*1.850*
Topo	*Order:3*	*-0.150*	*1.350*
Ont	Age	-0.166	0.786
**Clim**	**DD.10.Curr**	**0.063**	**0.399**
**Clim**	**DD.11.Curr**	**0.066**	**0.397**
**Clim**	**DD.08.Prev**	**0.027**	**0.341**
**Clim**	**DD.09.Prev**	**0.026**	**0.341**
**Clim**	**DD.10.Prev**	**0.025**	**0.341**
**Clim**	**DD.11.Prev**	**0.023**	**0.339**
**Clim**	**DD.12.Prev**	**0.018**	**0.336**
**Clim**	**DD.13.Prev**	**0.011**	**0.329**
**Clim**	**TXx.Sumr.Prev**	**-0.068**	**0.319**
**Clim**	**DD.18.Prev**	**-0.026**	**0.299**
**Clim**	**R.Wint.Prev**	**0.083**	**0.282**
**Clim**	**DD.20.Prev**	**-0.025**	**0.281**
**Clim**	**TNn.Sumr.Curr**	**0.056**	**0.186**
Loc	RP2	-0.082	0.823
Loc	RP3	0.077	0.795
Loc	RP3.PB2	-0.061	0.309

Tab a4-EWP2: Probability of male cones for Eastern white pine

	Variable	Coef	VIP
**Clim**	**TNn.02a.Prev**	**0.672**	**1.810**
**Clim**	**TNn.11a.Prev**	**0.444**	**0.946**
**Clim**	**TX.11a.Prev**	**0.401**	**0.850**
**Clim**	**TNn.02b.Prev**	**0.302**	**0.800**
**Clim**	**TN.03a.Prev**	**0.269**	**0.779**
**Clim**	**TNn.03a.Prev**	**0.265**	**0.756**
**Clim**	**TM.03a.Prev**	**0.263**	**0.749**
**Clim**	**TN.04b.Prev**	**0.360**	**0.719**
**Clim**	**TN.10b.Prev**	**0.304**	**0.719**
**Clim**	**TX.03a.Prev**	**0.249**	**0.698**
**Clim**	**R.02a.Curr**	**-0.337**	**0.689**
**Clim**	**TN.07b.Prev**	**0.336**	**0.673**
**Clim**	**R.02a.Prev**	**-0.205**	**0.579**
Ont	Autocor	0.246	0.548
**Clim**	**R.03a.Prev**	**0.260**	**0.547**
Ont	ASL	-0.152	0.410
Loc	RP2.PB1	-1.680	3.270
Loc	RP2.PB1.BSE1	-0.896	1.850
Loc	RP2.PB3.BNE2	-0.795	1.450
Loc	RP3.PB3.BN1	-0.785	1.440
Loc	RP2.PB2.BNO2	-0.730	1.360
Loc	RP2.PB1.ME3	-0.615	1.260
Loc	RP3.PB4.BN1	-0.658	1.230
Loc	RP2.PB1.BSO2	-0.579	1.190
Loc	RP1.PB2.BE2	-0.628	1.180
Loc	RP1.PB1.BSE2	-0.587	1.090
Loc	RP3.PB1.BO1	-0.586	1.090
Loc	RP3.PB5.BS1	-0.581	1.080
Loc	RP3.PB3.MSE5	-0.560	1.040
Loc	RP3.PB1.BE2	-0.551	1.030
Loc	RP3.PB3.BSE4	-0.552	1.030
Loc	RP1.PB2.BE1	-0.526	0.985
Loc	RP1.PB2.BO3	-0.485	0.908
Loc	RP2.PB1.MN4	-0.388	0.796
Loc	RP3.PB5.BS2	-0.426	0.786
Loc	RP1.PB2	-0.118	0.255
Loc	RP2.PB4	0.086	0.160
Loc	RP2.PB2.MSE1	0.073	0.144
Loc	RP2.PB2.MN4	0.069	0.124
Loc	RP2.PB4.MSO4	0.064	0.117
Loc	RP2.PB2	0.060	0.115
Loc	RP1.PB3.MN3	0.059	0.109
Loc	RP1.PB3.MSO4	0.051	0.095
Loc	RP1.PB5.MN4	0.048	0.090
Loc	RP1.PB1.ME3	0.045	0.089
Loc	RP2	0.049	0.063

Branches from mid-crown had a significantly higher signed VIP than low branches

Top vs low branches

	Variable	+/-VIP
**mid**	**RP2.PB2.MSE1**	**0.144**
**mid**	**RP2.PB2.MN4**	**0.124**
**mid**	**RP2.PB4.MSO4**	**0.117**
**mid**	**RP1.PB3.MN3**	**0.109**
**mid**	**RP1.PB3.MSO4**	**0.095**
**mid**	**RP1.PB5.MN4**	**0.090**
**mid**	**RP1.PB1.ME3**	**0.089**
low	RP3.PB5.BS2	-0.786
**mid**	**RP2.PB1.MN4**	**-0.796**
low	RP1.PB2.BO3	-0.908
low	RP1.PB2.BE1	-0.985
low	RP3.PB1.BE2	-1.030
low	RP3.PB3.BSE4	-1.030
**mid**	**RP3.PB3.MSE5**	**-1.040**
low	RP3.PB5.BS1	-1.080
low	RP1.PB1.BSE2	-1.090
low	RP3.PB1.BO1	-1.090
low	RP1.PB2.BE2	-1.180
low	RP2.PB1.BSO2	-1.190
low	RP3.PB4.BN1	-1.230
**mid**	**RP2.PB1.ME3**	**-1.260**
low	RP2.PB2.BNO2	-1.360
low	RP3.PB3.BN1	-1.440
low	RP2.PB3.BNE2	-1.450
low	RP2.PB1.BSE1	-1.850

Median VIP top: 0.09
Median VIP low: -1.09
Mann-Whitney (Wilcoxon) test
Mean rank top: 7.2
Mean rank low : 16.867
W = 17.0 P = 0.0014

Tab a4-EWP3: Length of male cones on AS for Eastern white pine

	Variable	Coef	VIP
Ont	Autocor	0.144	1.450
Ont	ASL	-0.150	1.270
**Clim**	**TNn.02b.Curr**	**-0.067**	**0.328**
**Clim**	**TNn.06b.Prev**	**0.058**	**0.270**
Loc	RP3.PB5	-0.107	1.090
Loc	RP2	0.091	0.798
Loc	RP2.PB2	0.142	0.743
Loc	RP2.PB3	-0.108	0.510

Tab a4-EWP4 Needle length (NL) for Eastern white pine

	Variable	Coef	VIP
Ont	ASL	0.200	1.970
Ont	Autocor	0.128	1.430
**Clim**	**TXx.Wint.Curr**	**-0.042**	**1.100**
**Clim**	**TXx.Sprg.Prev**	**0.042**	**1.100**
**Clim**	**R.Sprg.Curr**	**0.042**	**1.100**
**Clim**	**TXx.Sprg.Curr**	**-0.043**	**1.090**
**Clim**	**TX.Sumr.Prev**	**-0.041**	**1.090**
**Clim**	**TXx.Sumr.Curr**	**0.041**	**1.090**
**Clim**	**R.Sumr.Prev**	**0.043**	**1.090**
**Clim**	**TX.Sprg.Curr**	**-0.038**	**1.080**
**Clim**	**TNn.Sprg.Prev**	**0.037**	**1.080**
**Clim**	**TM.Sumr.Prev**	**-0.044**	**1.070**
**Clim**	**TX.Wint.Curr**	**-0.034**	**1.040**
**Clim**	**TX.Sprg.Prev**	**0.033**	**1.030**
Loc	RP3.PB2	-0.100	0.978
Loc	RP1.PB1	0.080	0.788
Loc	RP3.PB2.BS2	-0.065	0.630
Loc	RP2.PB5	-0.059	0.566
Loc	RP2.PB4	0.056	0.544
Loc	RP3.PB4.MN5	-0.050	0.497
Loc	RP2.PB4.BNE1	0.048	0.467
Loc	RP3.PB1.BO1	0.043	0.419
Loc	RP1.PB2	0.040	0.395
Loc	RP2.PB1.MN4	0.031	0.302
Loc	RP1.PB5.BSO1	-0.022	0.217

Tab a4-EWP5: Number of ramifications per AS for Eastern white pine

**Classes span**
0|≤1
1|≤2
2|≤3
3|≤4
4|≤5
5|≤6
6|≤7
7|≤8
8|≤9
9|≤10
10|≤11
11|13

	**Variable**	**Coef**	**VIP**
Ont	ASL	2.080	2.500
Ont	Autocor	0.541	1.580
Topo	*Vigour*	*0.252*	*0.844*
Topo	*Order:3*	*-0.169*	*0.629*
Ont	Age	-0.159	0.186
**Clim**	**TM.01a.Curr**	**-0.217**	**0.164**
**Clim**	**TXx.06b.Curr**	**-0.184**	**0.101**
**Clim**	**TN.12a.Prev**	**-0.200**	**0.098**
Loc	RP3.PB3	0.154	0.217
Loc	RP2.PB4	0.215	0.187
Loc	RP1.PB1	0.176	0.129
Loc	RP2.PB1	-0.195	0.100

Tab a4-EWP6: Number of cones per AS for Eastern white pine

Classes span
0|≤1
1|≤2
2|≤3
3|≤4

	Variable	Coef	VIP
Ont	ASL	0.436	0.697
Topo	*Vigour*	*0.112*	*0.442*
**Clim**	**TM.Sumr.Prev**	**-0.125**	**0.118**
**Clim**	**TN.Sumr.Prev**	**-0.128**	**0.116**
**Clim**	**TX.Sumr.Prev**	**-0.103**	**0.102**
Loc	RP2.PB4	-3.260	2.520
Loc	RP2.PB3	-3.000	2.370
Loc	RP2.PB1	-3.260	2.350
Loc	RP3.PB2	-2.850	2.220
Loc	RP1.PB2	-2.810	2.000
Loc	RP2.PB5.BSO1	-1.920	1.410
Loc	RP2.PB5.MS2	-1.870	1.380
Loc	RP2.PB4.MNO3	-1.320	1.340
Loc	RP2.PB1.BSE1	-1.360	1.330
Loc	RP2.PB3.MS4	-1.150	1.160
Loc	RP2.PB4.BNE1	-1.110	1.140
Loc	RP1.PB3.BNE1	-1.450	1.120
Loc	RP2.PB3.BNE2	-1.100	1.120
Loc	RP3.PB3.BN1	-1.460	1.110
Loc	RP1.PB4.MS5	-1.400	1.090
Loc	RP3.PB2.ME4	-1.070	1.070
Loc	RP2.PB2.BNO2	-1.370	0.971
Loc	RP3.PB4.BO2	-1.290	0.944
Loc	RP3.PB2.BN1	-0.971	0.909
Loc	RP2.PB1.ME3	-0.962	0.903
Loc	RP1.PB5.BSO1	-1.200	0.880
Loc	RP2.PB5.MN3	-1.160	0.880
Loc	RP3.PB4.BN1	-1.180	0.880
Loc	RP2.PB4.MSO4	-0.859	0.873
Loc	RP1.PB2.MS4	-0.880	0.855
Loc	RP2.PB1.BSO2	-0.913	0.855
Loc	RP2.PB2.MSE1	-1.170	0.855
Loc	RP1.PB2.BE2	-0.933	0.842
Loc	RP1.PB1.MO4	-1.120	0.836

Top vs low branches

	Variable	+/-VIP
**mid**	**RP2.PB5.MN4**	**0.163**
**mid**	**RP3.PB3.MSE5**	**0.144**
**mid**	**RP3.PB3.MN6**	**0.143**
**mid**	**RP3.PB5.MS5**	**0.115**
**mid**	**RP3.PB5.MN4**	**-0.430**
low	RP3.PB5.BS2	-0.606
**mid**	**RP2.PB1.MN4**	**-0.615**
low	RP1.PB2.BO3	-0.649
**mid**	**RP1.PB4.MN4**	**-0.673**
low	RP1.PB2.BE1	-0.704
**mid**	**RP3.PB1.MS4**	**-0.720**
low	RP2.PB4.BN2	-0.734
low	RP3.PB1.BE2	-0.734
low	RP3.PB3.BSE4	-0.734
**mid**	**RP3.PB2.ME6**	**-0.749**
**mid**	**RP1.PB1.ME3**	**-0.763**
**mid**	**RP1.PB3.MN3**	**-0.770**
**mid**	**RP2.PB2.MO3**	**-0.770**
low	RP3.PB5.BS1	-0.770
low	RP1.PB1.BSE2	-0.777
low	RP3.PB1.BO1	-0.777
**mid**	**RP3.PB1.MNE3**	**-0.777**
**mid**	**RP1.PB4.MN3**	**-0.797**
**mid**	**RP1.PB1.MSO1**	**-0.804**
**mid**	**RP2.PB2.MN4**	**-0.804**
**mid**	**RP1.PB4.MS2**	**-0.823**
**mid**	**RP1.PB1.MO4**	**-0.836**
**mid**	**RP2.PB3.MN5**	**-0.836**
**mid**	**RP2.PB3.MNE3**	**-0.836**

Loc	RP2.PB3.MN5	-0.841	0.836
Loc	RP2.PB3.MNE3	-0.930	0.836
Loc	RP3.PB2.BS2	-0.846	0.836
Loc	RP1.PB4.MS2	-1.110	0.823
Loc	RP1.PB1.MSO1	-1.100	0.804
Loc	RP2.PB2.MN4	-1.090	0.804
Loc	RP1.PB4.MN3	-1.070	0.797
Loc	RP1.PB1.BSE2	-1.050	0.777
Loc	RP3.PB1.BO1	-1.070	0.777
Loc	RP3.PB1.MNE3	-1.090	0.777
Loc	RP1.PB3.MN3	-1.030	0.770
Loc	RP2.PB2.MO3	-1.070	0.770
Loc	RP3.PB5.BS1	-1.070	0.770
Loc	RP1.PB1.ME3	-1.060	0.763
Loc	RP3.PB2.ME6	-0.750	0.749
Loc	RP2.PB4.BN2	-0.682	0.734
Loc	RP3.PB1.BE2	-0.961	0.734
Loc	RP3.PB3.BSE4	-0.987	0.734
Loc	RP3.PB1.MS4	-0.977	0.720
Loc	RP1.PB2.BE1	-0.714	0.704
Loc	RP1.PB4.MN4	-0.896	0.673
Loc	RP1.PB2.BO3	-0.674	0.649
Loc	RP2.PB1.MN4	-0.650	0.615
Loc	RP3.PB5.BS2	-0.797	0.606
Loc	RP3.PB5.MN4	-0.592	0.430
Loc	RP3.PB3	0.162	0.235
Loc	RP2.PB5	0.282	0.170
Loc	RP2.PB5.MN4	0.164	0.163
Loc	RP3.PB3.MSE5	0.110	0.144
Loc	RP3.PB3.MN6	0.121	0.143
Loc	RP3.PB5.MS5	0.097	0.115
Loc	RP1.PB5	0.112	0.100

low	RP3.PB2.BS2	-0.836
low	RP1.PB2.BE2	-0.842
**mid**	**RP1.PB2.MS4**	**-0.855**
low	RP2.PB1.BSO2	-0.855
**mid**	**RP2.PB2.MSE1**	**-0.855**
**mid**	**RP2.PB4.MSO4**	**-0.873**
low	RP1.PB5.BSO1	-0.880
**mid**	**RP2.PB5.MN3**	**-0.880**
low	RP3.PB4.BN1	-0.880
**mid**	**RP2.PB1.ME3**	**-0.903**
low	RP3.PB2.BN1	-0.909
low	RP3.PB4.BO2	-0.944
low	RP2.PB2.BNO2	-0.971
**mid**	**RP3.PB2.ME4**	**-1.070**
**mid**	**RP1.PB4.MS5**	**-1.090**
low	RP3.PB3.BN1	-1.110
low	RP1.PB3.BNE1	-1.120
low	RP2.PB3.BNE2	-1.120
low	RP2.PB4.BNE1	-1.140
**mid**	**RP2.PB3.MS4**	**-1.160**
low	RP2.PB1.BSE1	-1.330
**mid**	**RP2.PB4.MNO3**	**-1.340**
**mid**	**RP2.PB5.MS2**	**-1.380**
low	RP2.PB5.BSO1	-1.410

Median VIP top:-0.804
Median VIP low: -0.855
Mann-Whitney (Wilcoxon) test
Mean rank top: 24.267
Mean rank low : 30.565
W = 264.5   P = 0.151

No significant VIP differences between middle and low branches

Tab a4-EWP7: Polyclism for Eastern white pine

Classes span	VIP
0|≤1	
1|2	

	Variable	Coef	VIP
Ont	ASL	0.553	0.977
Topo	*Vigour*	*0.421*	*0.809*
**Clim**	**TX.04.Curr**	**0.199**	**0.572**
**Clim**	**TM.04.Curr**	**0.169**	**0.547**
**Clim**	**TN.04.Curr**	**0.139**	**0.502**
Topo	*Order:3*	*-0.219*	*0.455*
**Clim**	**TXx.04.Prev**	**0.134**	**0.448**
**Clim**	**TNn.04.Curr**	**0.093**	**0.430**
**Clim**	**TNn.06.Curr**	**0.257**	**0.421**
**Clim**	**TNn.11.Prev**	**0.119**	**0.413**
**Clim**	**TNn.03.Prev**	**-0.225**	**0.389**
**Clim**	**TXx.04.Curr**	**0.098**	**0.330**
**Clim**	**R.11.Prev**	**-0.073**	**0.305**
**Clim**	**R.01.Curr**	**-0.065**	**0.304**
**Clim**	**TNn.04.Prev**	**0.131**	**0.293**
**Clim**	**TN.04.Prev**	**0.215**	**0.291**
**Clim**	**TNn.02.Curr**	**0.019**	**0.269**
**Clim**	**R.04.Prev**	**0.062**	**0.268**
**Clim**	**TX.11.Curr**	**0.092**	**0.252**
**Clim**	**TXx.07.Prev**	**-0.027**	**0.234**
**Clim**	**R.05.Curr**	**-0.040**	**0.232**
**Clim**	**TM.11.Curr**	**0.127**	**0.231**
**Clim**	**TN.05.Prev**	**-0.103**	**0.225**
**Clim**	**TM.04.Prev**	**0.107**	**0.224**
**Clim**	**R.06.Prev**	**-0.052**	**0.218**
**Clim**	**TX.08.Prev**	**-0.021**	**0.212**
**Clim**	**TX.07.Prev**	**-0.010**	**0.206**
**Clim**	**R.07.Prev**	**0.040**	**0.200**
**Clim**	**TN.06.Curr**	**0.130**	**0.195**
**Clim**	**TM.07.Prev**	**-0.028**	**0.188**
**Clim**	**TX.04.Prev**	**0.060**	**0.183**
**Clim**	**TN.11.Curr**	**0.149**	**0.181**
**Clim**	**TXx.09.Curr**	**0.041**	**0.169**
**Clim**	**R.10.Curr**	**0.031**	**0.160**
**Clim**	**TX.12.Prev**	**-0.088**	**0.155**
**Clim**	**TM.12.Prev**	**-0.068**	**0.149**
**Clim**	**TN.12.Prev**	**-0.055**	**0.142**
**Clim**	**TN.09.Prev**	**0.033**	**0.141**

Loc	RP2	-2.190	3.920
Loc	RP3	2.180	3.890
Loc	RP1	-2.080	3.690
Loc	RP2.PB5	-1.220	2.230
Loc	RP2.PB4	-1.070	1.950
Loc	RP2.PB3	-0.987	1.830
Loc	RP2.PB1	-1.040	1.820
Loc	RP1.PB3	-0.981	1.800
Loc	RP1.PB5	-0.966	1.770
Loc	RP3.PB2	-1.060	1.720
Loc	RP2.PB2	-0.941	1.680
Loc	RP1.PB4	-0.916	1.650
Loc	RP1.PB1	-0.887	1.590
Loc	RP1.PB2	-0.878	1.550
Loc	RP3.PB1	-0.970	1.510
Loc	RP2.PB5.BSO1	-0.556	1.090
Loc	RP2.PB5.MS2	-0.542	1.060
Loc	RP2.PB4.MNO3	-0.522	1.040
Loc	RP2.PB1.BSE1	-0.522	1.020
Loc	RP2.PB3.MS4	-0.459	0.897
Loc	RP1.PB5.BN2	-0.455	0.881
Loc	RP2.PB4.BNE1	-0.447	0.881
Loc	RP1.PB3.BNE1	-0.454	0.868
Loc	RP2.PB3.BNE2	-0.441	0.864
Loc	RP1.PB3.MSO4	-0.449	0.860
Loc	RP3.PB3.BN1	-0.544	0.857
Loc	RP1.PB4.MS5	-0.436	0.843
Loc	RP3.PB2.ME4	-0.498	0.830
Loc	RP2.PB2.BNO2	-0.430	0.807
Loc	RP3.PB4.BO2	-0.502	0.786
Loc	RP3.PB2.BN1	-0.455	0.757
Loc	RP2.PB1.ME3	-0.395	0.751
Loc	RP3.PB4.BN1	-0.439	0.679
Loc	RP1.PB5.BSO1	-0.357	0.678
Loc	RP2.PB5.MN3	-0.348	0.678
Loc	RP2.PB4.MSO4	-0.342	0.673
Loc	RP1.PB3.MS2	-0.349	0.669
Loc	RP1.PB2.MS4	-0.344	0.659
Loc	RP1.PB5.MS3	-0.354	0.659
Loc	RP2.PB1.BSO2	-0.346	0.659
Loc	RP2.PB2.MSE1	-0.346	0.659
Loc	RP1.PB2.BE2	-0.352	0.650
Loc	RP1.PB5.MN4	-0.333	0.650
Loc	RP1.PB1.MO4	-0.328	0.645
Loc	RP2.PB3.MN5	-0.322	0.645

Top vs low branches

	**Variable**	**+/-VIP**
**mid**	**RP3.PB3.MN6**	**0.117**
**mid**	**RP3.PB5.MN4**	**-0.332**
**mid**	**RP3.PB4.MN5**	**-0.468**
low	RP3.PB5.BS2	-0.468
**mid**	**RP2.PB1.MN4**	**-0.474**
low	RP1.PB2.BO3	-0.500
**mid**	**RP1.PB4.MN4**	**-0.519**
low	RP1.PB2.BE1	-0.543
**mid**	**RP3.PB1.MS4**	**-0.555**
**mid**	**RP3.PB4.MS3**	**-0.556**
low	RP2.PB4.BN2	-0.566
low	RP3.PB1.BE2	-0.567
low	RP3.PB3.BSE4	-0.567
**mid**	**RP3.PB2.ME6**	**-0.578**
**mid**	**RP2.PB5.MN4**	**-0.583**
**mid**	**RP1.PB1.ME3**	**-0.588**
**mid**	**RP1.PB3.MN3**	**-0.593**
**mid**	**RP2.PB2.MO3**	**-0.593**
low	RP3.PB5.BS1	-0.595
low	RP3.PB1.BO1	-0.599
**mid**	**RP3.PB1.MNE3**	**-0.599**
low	RP1.PB1.BSE2	-0.599
**mid**	**RP1.PB4.MN3**	**-0.614**
**mid**	**RP1.PB1.MSO1**	**-0.620**
**mid**	**RP2.PB2.MN4**	**-0.620**
**mid**	**RP1.PB4.MS2**	**-0.635**
**mid**	**RP1.PB1.MO4**	**-0.645**
**mid**	**RP2.PB3.MN5**	**-0.645**
**mid**	**RP2.PB3.MNE3**	**-0.645**
low	RP3.PB2.BS2	-0.645
low	RP1.PB2.BE2	-0.650
**mid**	**RP1.PB5.MN4**	**-0.650**
**mid**	**RP1.PB2.MS4**	**-0.659**
**mid**	**RP1.PB5.MS3**	**-0.659**
low	RP2.PB1.BSO2	-0.659
**mid**	**RP2.PB2.MSE1**	**-0.659**
**mid**	**RP1.PB3.MS2**	**-0.669**
**mid**	**RP2.PB4.MSO4**	**-0.673**
low	RP1.PB5.BSO1	-0.678
**mid**	**RP2.PB5.MN3**	**-0.678**
**mid**	**RP3.PB4.BN1**	**-0.679**
**mid**	**RP2.PB1.ME3**	**-0.751**
low	RP3.PB2.BN1	-0.757

Loc	RP2.PB3.MNE3	-0.345	0.645
Loc	RP3.PB2.BS2	-0.380	0.645
Loc	RP1.PB4.MS2	-0.323	0.635
Loc	RP1.PB1.MSO1	-0.322	0.620
Loc	RP2.PB2.MN4	-0.320	0.620
Loc	RP1.PB4.MN3	-0.314	0.614
Loc	RP3.PB1.BO1	-0.370	0.599
Loc	RP3.PB1.MNE3	-0.367	0.599
Loc	RP1.PB1.BSE2	-0.312	0.599
Loc	RP3.PB5.BS1	-0.395	0.595
Loc	RP1.PB3.MN3	-0.303	0.593
Loc	RP2.PB2.MO3	-0.313	0.593
Loc	RP1.PB1.ME3	-0.307	0.588
Loc	RP2.PB5.MN4	-0.304	0.583
Loc	RP3.PB2.ME6	-0.349	0.578
Loc	RP3.PB1.BE2	-0.346	0.567
Loc	RP3.PB3.BSE4	-0.369	0.567
Loc	RP2.PB4.BN2	-0.280	0.566
Loc	RP3.PB4.MS3	-0.369	0.556
Loc	RP3.PB1.MS4	-0.346	0.555
Loc	RP1.PB2.BE1	-0.279	0.543
Loc	RP1.PB4.MN4	-0.276	0.519
Loc	RP1.PB2.BO3	-0.264	0.500
Loc	RP2.PB1.MN4	-0.253	0.474
Loc	RP3.PB4.MN5	-0.311	0.468
Loc	RP3.PB5.BS2	-0.309	0.468
Loc	RP3.PB5.MN4	-0.226	0.332
Loc	RP3.PB5	0.128	0.284
Loc	RP3.PB3	0.114	0.247
Loc	RP3.PB3.MN6	0.055	0.117

low	RP3.PB4.BO2	-0.786
low	RP2.PB2.BNO2	-0.807
**mid**	**RP3.PB2.ME4**	**-0.830**
**mid**	**RP1.PB4.MS5**	**-0.843**
low	RP3.PB3.BN1	-0.857
**mid**	**RP1.PB3.MSO4**	**-0.860**
low	RP2.PB3.BNE2	-0.864
low	RP1.PB3.BNE1	-0.868
low	RP1.PB5.BN2	-0.881
low	RP2.PB4.BNE1	-0.881
**mid**	**RP2.PB3.MS4**	**-0.897**
low	RP2.PB1.BSE1	-1.020
**mid**	**RP2.PB4.MNO3**	**-1.040**
**mid**	**RP2.PB5.MS2**	**-1.060**
low	RP2.PB5.BSO1	-1.090

Median VIP top:-0.645
Median VIP low: -0.659
Mann-Whitney (Wilcoxon) test
Mean rank top: 27.486
Mean rank low : 32.565
W =333.5 P = 0.276

No significant VIP differences between middle and low branches

Tab a4-JP1: Annual shoot length (ASL) for Jack pine

	Variable	Coef	VIP
Ont	Autocor	0.344	3.200
Topo	*Vigour*	*0.260*	*2.240*
Topo	*Order:2*	*0.127*	*1.820*
Topo	*Order:3*	*-0.127*	*1.810*
Ont	Age	-0.045	0.518
**Clim**	**TXx.02.Prev**	**0.054**	**0.430**
**Clim**	**TNn.07.Prev**	**-0.039**	**0.352**
**Clim**	**R.Sumr.11.Prev**	**0.037**	**0.267**
Loc	GJ2.PG3	0.101	1.030
Loc	GJ3.PG1	-0.091	0.878
Loc	GJ3.PG4.MNE3	-0.033	0.677
Loc	GJ3	-0.061	0.652
Loc	GJ3.PG4	-0.042	0.644
Loc	GJ2.PG2	0.072	0.631
Loc	GJ1.PG2.BN1	-0.049	0.548
Loc	GJ2.PG1.HE7	-0.041	0.515
Loc	GJ3.PG2	0.050	0.496
Loc	GJ1.PG5.BE1	-0.042	0.470
Loc	GJ2.PG4	-0.059	0.434
Loc	GJ3.PG3.BS1	-0.031	0.421
Loc	GJ2.PG3.HE1	0.029	0.354
Loc	GJ1.PG4.BN2	-0.020	0.342
Loc	GJ3.PG5.BN2	-0.025	0.330
Loc	GJ1.PG4	-0.030	0.267
Loc	GJ3.PG5	0.029	0.266
Loc	GJ2.PG2.HO6	0.022	0.182
Loc	GJ1.PG5.HO6	0.024	0.153
Loc	GJ1.PG2.HS4	0.017	0.112

Top vs low branches

	Variable	+/-VIP
**top**	**GJ2.PG3.HE1**	**0.354**
**top**	**GJ2.PG2.HO6**	**0.182**
**top**	**GJ1.PG5.HO6**	**0.153**
**top**	**GJ1.PG2.HS4**	**0.112**
low	GJ3.PG5.BN2	-0.330
low	GJ1.PG4.BN2	-0.342
low	GJ3.PG3.BS1	-0.421
low	GJ1.PG5.BE1	-0.470
**top**	**GJ2.PG1.HE7**	**-0.515**
low	GJ1.PG2.BN1	-0.548
low	GJ3.PG4.MNE3	-0.677

	VIP top	VIP low
Mean	0.057	-0.465
	Variance analysis	
t = 3.550	P = 0.006	

Top branches had a significantly higher signed VIP than low branches

Tab a4-JP2: Probability of male cones for Jack pine

	Variable	Coef	VIP
Topo	Order:1	-0.811	1.080
**Clim**	**TXx.02.Prev**	**-0.347**	**0.945**
Ont	*Autocor*	*0.638*	*0.917*
**Clim**	**DD.07.Prev**	**0.284**	**0.725**
**Clim**	**R.05.Curr**	**0.214**	**0.680**
**Clim**	**TNn.08.Prev**	**0.275**	**0.661**
**Clim**	**TXx.02.Curr**	**-0.315**	**0.655**
**Clim**	**R.11.Prev**	**-0.310**	**0.652**
Ont	*ASL*	*-0.432*	*0.593*
**Clim**	**TNn.01.Curr**	**-0.321**	**0.585**
**Clim**	**DD.04.Curr**	**0.235**	**0.557**
**Clim**	**DD.05.Curr**	**0.219**	**0.555**
**Clim**	**DD.03.Curr**	**0.240**	**0.550**
**Clim**	**DD.02.Curr**	**0.232**	**0.545**
**Clim**	**DD.01.Curr**	**0.220**	**0.536**
**Clim**	**TXx.04.Prev**	**0.236**	**0.531**
**Clim**	**DD.00.Curr**	**0.217**	**0.522**
**Clim**	**R.08.Prev**	**0.241**	**0.513**
**Clim**	**TXx.04.Curr**	**0.365**	**0.490**
**Clim**	**TN.02.Prev**	**0.109**	**0.415**
**Clim**	**TN.04.Prev**	**0.223**	**0.380**
**Clim**	**R.03.Curr**	**-0.280**	**0.347**
**Clim**	**TXx.11.Prev**	**0.221**	**0.308**
**Clim**	**TXx.08.Prev**	**0.126**	**0.307**
**Clim**	**TN.06.Prev**	**0.553**	**0.301**
**Clim**	**TM.04.Prev**	**0.189**	**0.295**
**Clim**	**TM.06.Prev**	**0.529**	**0.292**
**Clim**	**TNn.12.Prev**	**-0.217**	**0.205**
**Clim**	**TX.04.Prev**	**0.144**	**0.189**
**Clim**	**TN.06.Curr**	**0.187**	**0.164**
**Clim**	**TNn.06.Prev**	**0.241**	**0.010**
Loc	GJ2.PG1	-3.020	4.150
Loc	GJ2.PG1.B01	-1.290	2.210
Loc	GJ2.PG4.HE3	-1.550	2.020
Loc	GJ2.PG1.HE7	-1.190	2.000
Loc	GJ2.PG3.BE5	-1.100	1.430
Loc	GJ3	0.236	0.326
Loc	GJ3.PG4	0.255	0.314
Loc	GJ2.PG2	0.182	0.256
Loc	GJ2.PG2.BE2	0.163	0.237
Loc	GJ2.PG4.BO1	0.105	0.149
Loc	GJ1.PG4.BN2	0.142	0.141

Tab a4-JP3: Length of male cones on AS for Jack pine

	Variable	Coef
Ont	ASL	0.736
**Clim**	**TN.08a.Prev**	**0.174**

Tab a4-JP4 Needle length for Jack pine

	Variable	Coef	VIP
Ont	Autocor	0.379	1.630
Ont	ASL	0.312	1.270
**Clim**	**TM.Sumr.Curr**	**0.121**	**0.543**
Loc	GJ2.PG2	0.065	0.573
Loc	GJ2.PG1	-0.073	0.352

Tab a4-JP5 Number of ramifications on AS for Jack pine

Classes span
0|≤1
1|≤2
2|≤3
3|≤4
4|≤5
5|≤6
6|≤7
7|≤8
8|10

	Variable	Coef	VIP
Ont	ASL	2.680	2.870
Topo	*Order:2*	*-3.470*	*1.710*
Topo	*Order:3*	*-4.080*	*1.700*
**Clim**	**TN.08b.Curr**	**-0.355**	**0.388**
**Clim**	**R.11a.Prev**	**0.417**	**0.372**
**Clim**	**Tn.08a.Prev**	**-0.306**	**0.340**
**Clim**	**DD.07.Prev**	**-0.233**	**0.248**
**Clim**	**DD.04.Prev**	**-0.244**	**0.247**
**Clim**	**DD.05.Prev**	**-0.241**	**0.247**
**Clim**	**TM.07b.Curr**	**0.185**	**0.140**
Loc	GJ1.PG5.BE1	-0.285	0.390
Loc	GJ3.PG5	0.262	0.281
Loc	GJ3.PG3	-0.221	0.211
Loc	GJ2.PG3.BE5	-0.307	0.201
Loc	GJ1.PG2	-0.224	0.140

Tab a4-JP6: Number of cones per AS for Jack pine

Classes span
0|≤1
1|≤2
2|≤3
3|≤4
4|≤5
5|6

	Variable	Coef	VIP
Ont	ASL	1.560	1.380
Topo	*Order:3*	*-0.299*	*0.767*
Topo	*Order:2*	*0.354*	*0.734*
Topo	*Order:1*	*0.386*	*0.201*
**Clim**	**TM.02.Curr**	**-0.099**	**0.099**
**Clim**	**R.12.Prev**	**-0.104**	**0.099**
**Clim**	**R.09.Prev**	**0.166**	**0.090**
**Clim**	**TM.03.Prev**	**0.147**	**0.082**
**Clim**	**TM.12.Prev**	**0.184**	**0.074**
**Clim**	**TX.03.Prev**	**0.090**	**0.050**
Loc	GJ3.PG1.HN4	-1.770	1.480
Loc	GJ3.PG4.MNE3	-1.760	1.480
Loc	GJ1.PG4.BN2	-1.820	1.470
Loc	GJ3.PG4.BSO1	-1.850	1.430
Loc	GJ1.PG2.BN1	-1.580	1.370
Loc	GJ1.PG5.BE1	-1.670	1.370
Loc	GJ3.PG5.BN2	-1.650	1.280
Loc	GJ1.PG3.BN3	-1.610	1.170
Loc	GJ3.PG1.BN1	-1.490	1.120
Loc	GJ1.PG3.HN4	-1.520	1.030
Loc	GJ1.PG1.HNE5	-1.370	1.010
Loc	GJ2.PG3.BE5	-1.410	0.903
Loc	GJ1	-0.536	0.225

Tab a4-JP7: Polycyclism for Jack pine

	Classes span		
	1|≤2		
	2|3		

	Variable	Coef	VIP
Ont	ASL	4.130	2.030
Topo	*Order:3*	*-0.696*	*0.678*
**Clim**	**TNn.04.Curr**	**-0.187**	**0.095**
Loc	GJ3.PG1.HN4	-1.710	1.160
Loc	GJ3.PG4.MNE3	-1.690	1.160
Loc	GJ1.PG4.BN2	-1.820	1.130
Loc	GJ1.PG2.BN1	-1.560	1.080
Loc	GJ1.PG5.BE1	-1.700	1.050
Loc	GJ3.PG5.BN2	-1.590	0.916
Loc	GJ1.PG3.BN3	-1.600	0.894
Loc	GJ3.PG1.BN1	-1.420	0.864
Loc	GJ1.PG3.HN4	-1.500	0.793
Loc	GJ1.PG1.HNE5	-1.330	0.772
Loc	GJ2.PG3.BE5	-1.340	0.700
Loc	GJ1	-0.476	0.289
